# Three Members of the 6-cys Protein Family of *Plasmodium* Play a Role in Gamete Fertility

**DOI:** 10.1371/journal.ppat.1000853

**Published:** 2010-04-08

**Authors:** Melissa R. van Dijk, Ben C. L. van Schaijk, Shahid M. Khan, Maaike W. van Dooren, Jai Ramesar, Szymon Kaczanowski, Geert-Jan van Gemert, Hans Kroeze, Hendrik G. Stunnenberg, Wijnand M. Eling, Robert W. Sauerwein, Andrew P. Waters, Chris J. Janse

**Affiliations:** 1 Laboratory for Parasitology, Leiden University Medical Centre, Leiden, The Netherlands; 2 Department of Medical Microbiology, Radboud University Nijmegen Medical Center, Nijmegen, The Netherlands; 3 Institute of Biochemistry and Biophysics, Polish Academy of Sciences, Warszawa, Poland; 4 Department of Molecular Biology, NCMLS, University of Nijmegen, Nijmegen, The Netherlands; 5 Division of Infection and Immunity, Institute of Biomedical Life Sciences & Wellcome Centre for Molecular Parasitology, Glasgow Biomedical Research Centre, University of Glasgow, Glasgow, Scotland; Albert Einstein College of Medicine, United States of America

## Abstract

The process of fertilization is critically dependent on the mutual recognition of gametes and in *Plasmodium*, the male gamete surface protein P48/45 is vital to this process. This protein belongs to a family of 10 structurally related proteins, the so called 6-cys family. To identify the role of additional members of this family in *Plasmodium* fertilisation, we performed genetic and functional analysis on the five members of the 6-cys family that are transcribed during the gametocyte stage of *P. berghei*. This analysis revealed that in addition to P48/45, two members (P230 and P47) also play an essential role in the process of parasite fertilization. Mating studies between parasites lacking P230, P48/45 or P47 demonstrate that P230, like P48/45, is a male fertility factor, consistent with the previous demonstration of a protein complex containing both P48/45 and P230. In contrast, disruption of P47 results in a strong reduction of female fertility, while males remain unaffected. Further analysis revealed that gametes of mutants lacking expression of *p48/45* or *p230* or *p47* are unable to either recognise or attach to each other. Disruption of the paralog of *p230*, *p230p*, also specifically expressed in gametocytes, had no observable effect on fertilization. These results indicate that the *P. berghei* 6-cys family contains a number of proteins that are either male or female specific ligands that play an important role in gamete recognition and/or attachment. The implications of low levels of fertilisation that exist even in the absence of these proteins, indicating alternative pathways of fertilisation, as well as positive selection acting on these proteins, are discussed in the context of targeting these proteins as transmission blocking vaccine candidates.

## Author Summary

Sexual reproduction for malaria parasites is an essential process and is necessary for parasite transmission between hosts. Fertilisation between female and male gametes occurs in the midgut of the mosquito and proteins on the surface of gametes are principle targets in transmission blocking strategies. Despite their importance, relatively little is known about the malaria proteins involved in fertilisation. In this study we show that two gamete proteins, one expressed on the surface of males, the other on the surface of females, have important roles in the mutual recognition and attachment of gametes. Mutant parasites that lack the presence of these surface proteins show a strong reduction in fertility. Comparison of these gamete surface proteins in different malaria parasites showed that these proteins are evolving rapidly either across their length or at discreet regions/domains. We found, that despite the drastic reduction in zygote formation, low levels of fertilisation can still occur in the absence of these surface proteins, indicating that gametes can use alternative proteins to recognize each other. Both genetic variation of gamete surface proteins and the presence of different fertilisation pathways have important implications for transmission blocking vaccines targeting gamete surface proteins.

## Introduction

Sexual reproduction is an obligate process in the *Plasmodium* life cycle and is required for transmission of the parasites between the vertebrate and mosquito hosts. The sexual phase is initiated by the formation of male and female cells (gametocytes) in the blood of the vertebrate host. Gametocytes are the precursors to the haploid male and female gametes that are produced in the mosquito midgut where fertilisation takes place. Successful fertilisation requires an ordered series of gamete-gamete interactions, specifically, the recognition of and adhesion to the female gamete by the motile male gamete, followed by a cascade of signalling events resulting from the fusion of the two gametes.

Despite their fundamental importance, relatively little is known about gamete receptors/ligands and their involvement in the process of gamete interactions of eukaryotes [1], [2], which is partly due to their rapid evolution and species-specific characteristics [3]. In *Plasmodium* the involvement of two gamete specific surface proteins P48/45 and HAP2/GCS1 has been demonstrated in male fertility and these proteins are to date the only known proteins with a demonstrable role in gamete-gamete interaction [4], [5], [6]. Parasites lacking P48/45 produce male gametes that fail to attach to fertile female gametes [4] while male gametes lacking of HAP2/GCS1 do attach to females, but they do not fuse due to an absence of membrane fusion between the two gametes [5]. P48/45 is one member of a family of proteins encoded within the genome of *Plasmodium* and this family is characterised by domains of roughly 120 amino acids in size that contain six positionally conserved cysteines (6-cys). The 6-cys family of proteins appears to be Apicomplexan specific and has a predicted relationship to the SAG proteins in *Toxoplasma gondii* [7], [8], [9], [10], [11]. Ten members of the 6-cys family have been identified. Most members are expressed in a discrete stage-specific manner in gametocytes, sporozoites or merozoites [8], [12], [13], [14], [15], [16]. The surface location of members of this family and their expression in gametes or in invasive stages (sporozoites and merozoites) suggests that they function in cell-cell interactions as has been shown for P48/45 in gamete adhesion. In addition to P48/45, five other 6-cys genes are transcribed in gametocytes, three of which (*p230*, *p230p* and *p47*) are exclusively expressed in the gamete stages of the malaria parasite [4], [8], [10], [12], [16], [17], [18], [19], indicating that these members of the gene family may also play a role in the process of gamete recognition and fertilisation. Indeed specific antibodies against the sexual stages of the human parasite *Plasmodium falciparum*, P48/45 and P230 can prevent zygote formation and thus block transmission of the parasite [19], [20], [21], [22], [23], [24], [25], [26]. Interestingly, *P. falciparum* mutants lacking P230 expression produce male gametes that fail to attach to erythrocytes resulting in a reduced formation of the characteristic ‘exflagellation centres’ and reduced oocyst formation in mosquitoes [27]. In order to investigate the role of the 6-cys proteins in parasite fertilisation we performed genetic and functional analysis on the five 6-cys proteins that are expressed in gametocytes. In this paper, we present evidence that in addition to P48/45, two 6-cys members (P230 and P47) also have an essential role in parasite fertilization. Interestingly, in *P. falciparum* evidence has been published that P48/45, P47 and P230 are under positive selection resulting in non-neutral sequence polymorphisms [28], [29], [30], [31]. By sequence analysis, we provide evidence that these three 6-cys proteins are undergoing strong but different rates of positive selection, either as a consequence sexual-selection driven by the competition between gametes or from natural selection exerted by the adaptive immune system of the host on proteins expressed in gametocytes.

## Materials and Methods

### Parasites

The gametocyte-producer clone cl15cy1 (HP) of *P. berghei* ANKA was used as the reference parasite line [32]. In addition, the following mutant lines of the ANKA strain were used: 2.33, a non-gametocyte producer (NP) line [33] and 137cl8 (RMgm-15, www.pberghei.eu), a mutant lacking expression of P48/45 [4].

### Generation of mutants deficient in expressing 6-cys family members

To disrupt genes encoding different members of the 6-cys family, we constructed a number replacement constructs using plasmid pL0001 (www.mr4.com) which contains the pyrimethamine resistant *Toxoplasma gondii* (*tg*) *dhfr/ts* as a selectable-marker cassette (SC). Target sequences for homologous recombination were PCR amplified from *P. berghei* genomic DNA (ANKA, cl15cy1) using primers specific for the 5′ or 3′ end of the different 6-cys genes (see Table S1 for the sequence of the different primers). The PCR–amplified target sequences were cloned in plasmid pL0001 either upstream or downstream of the SC to allow for integration of the construct into the genomic target sequence by homologous recombination. DNA constructs used for transfection were obtained after digestion of the replacement constructs with the appropriate restriction enzymes (Table S1). Replacement constructs pL1138 (*p47*) and pL0123 (*p36*), were constructed using replacement plasmid pD_B._D_T∧H._D_B_ [34] and plasmid pL0121 (*p47*&*48/45*) was constructed in the previously described replacement plasmid for disruption of *pb48/45* (plasmid p54 is renamed here to pL1137; [4]). This plasmid was made by exchanging the 5′ *pb48/45* targeting sequence with the 5′ targeting sequence of *pb47*. The *p230p*II replacement construct pL0120 is a derivative of plasmid pL0016 [35] containing the *tgdhfr-ts* SC, *gfp* (under control of the *pbeef1aa* promoter and 3′UTR of *pbdhfr/ts*) and *p230p* 5′ and 3′ targeting sequences [36]. Transfection, selection and cloning of mutant parasite lines were performed as described [32], [37] using *P. berghei* ANKA cl15cy1 as the parent reference line. For all mutants with an observable phenotype, mutants were generated and selected in two independent transfection experiments (Table S1). Of each transfection experiment we selected one cloned line for further genotype and phenotype analysis. Correct integration of the construct into the genome of mutant parasites was analysed by standard PCR analysis and Southern blot analysis of digested genomic DNA or of FIGE separated chromosomes [32]. PCR analysis on genomic DNA was performed using specific primers to amplify either part of the wild type locus (primers WT1 and 2) or the disrupted locus (primers INT1 and 2). See Table S2 for the sequence of these primers.

### Analysis of expression by Northern and Western analysis

Total RNA was isolated from the different blood stage parasites of the gametocyte-producer clone cl15cy1 of *P. berghei* ANKA (HP), the non-gametocyte producer line 2.33 (NP) and the different mutant lines according to standard methods. To determine stage-specific transcription of the 6-cys family members, Northern blots containing RNA from different blood stages were hybridised with different gene specific probes, which were PCR-amplified using the primers shown in Table S2 (primer pairs WT1+ 2). To detect expression of the P48/45 protein we used polyclonal antiserum raised against recombinant *P. berghei* P48/45 as described [4]. For detection of P47 we generated the following polyclonal antiserum; a fragment of the *Pb47* ORF (encoding amino acids 80–411) was PCR-amplified using primers L964 and L965 (Table S2) and cloned into the *Nde*I/*Bam*HI sites of the expression vector pET-15b (Novagen) providing an N-terminal 6-Histidine tag. Polyclonal antiserum was raised in New Zealand rabbits by injection of 200 µg of gel-purified recombinant protein. Boosting was carried out subcutaneously with 3-weeks intervals using 200 µg protein in incomplete Freund's adjuvant. Serum (P47) obtained 2 weeks after the third boost was immuno-purified on immobilised purified recombinant P47. To detect P48/45 and P47 in the different mutant lines, total protein samples of purified gametocytes were fractionated on non-reducing 10% SDS polyacrylamide gels.

### Phenotype analysis of parasite lines lacking expression of 6-cys gene family members

The fertility of wild type and mutant gamete populations was analysed by standard *in vitro* fertilisation and ookinete maturation assays [4], [17] from highly pure gametocyte populations [38]. The fertilisation rate of gametes is defined as the percentage of female gametes that develop into mature ookinetes determined by counting female gametes and mature ookinetes in Giemsa stained blood smears 16–18 hours after *in vitro* induction of gamete formation. Fertility of individual sexes (macro- and micro-gametes) was determined by *in vitro* cross-fertilisation studies in which gametes are cross-fertilised with gametes of lines that produce only fertile male (Δ*p47*; 270cl1) or only fertile female gametes (Δ*p48/45*; 137cl1 [4], [17], [39]. All fertilisation and ookinete maturation assays were done in triplicate on multiple occasions in independent experiments. *In vivo* ookinete, oocyst and salivary gland sporozoite production of the mutant parasites were determined by performing standard mosquito infections by feeding of *Anopheles stephensi* mosquitoes on infected mice [40]. Oocyst numbers and salivary gland sporozoites were counted at 7–10 days and 21–22 days respectively after mosquito infection. For counting sporozoites, salivary glands from 10 mosquitoes were dissected and homogenized in a homemade glass grinder in 1000µl of PBS pH 7.2 and sporozoites were counted in a Bürker-Türk counting chamber using phase-contrast microscopy [41]. Infectivity of sporozoites was determined by infecting mice through bites of 25–30 infected mosquitoes at day 21–25 after mosquito infection.

The formation of exflagellation centres (i.e. male gamete interactions with red blood cells) was determined by adding 10µl of infected tail blood to 100–300 µl of standard ookinete culture medium pH 8.2 to induce gamete formation. Ten minutes after induction of gamete formation a droplet of 5–10 µl was placed on a cover slip and analysed under a standard light microscope (40× magnification) as a hanging-drop using a well slide. When red blood cells were settled in a monolayer, the number of exflagellating male gametocytes was counted that form or did not form exflagellation centres. An exflagellation centre is defined as an exflaggelating male gametocyte with more than four tightly associated red blood cells [27]. The formation of exflagellation centres was performed using tail blood collected at day 6 or 7 from mice that were infected with 10^5^ parasites without treatment with phenylhydrazine. For quantification of male-female interactions tail blood was collected from phenylhydrazine-treated mice with high numbers of gametocytes [42]. Tail blood (10µl) was collected at gametocytemias ranging between 4–8% and added to 100µl of standard ookinete culture medium pH 8.2 to induce gamete formation. Ten minutes after induction of gamete formation, the cell suspension was placed in a Bürker-Türk counting chamber and during a period of twenty minutes the male-female interactions were scored using a phase-contrast light microscope at a 40× magnification. Attachments of males to females were scored if the male had active (attachment-) interactions with the female for more than 3 seconds. Penetration of a female by the male gamete was scored as a fertilisation event.

### Polymorphisms and sequence divergence of the *Plasmodium* 6-cys genes

Pairwise alignments were generated between the orthologous sequences of *p48/45*, *p47* and *p230* genes in *P. berghei*, *P. yoelii* and *P. chabaudi*; sequences were obtained from PlasmoDB (http://www.plasmodb.org version 6.1; see Table S3 for the accession numbers of the 6-cys gene family members). Complete gene sequences for a number of these genes were obtained from the Sanger Institute (A. Pain, personal communication). Maximum-likelihood estimates of rates of non-synonymous substitution (dN) and synonymous substitution (dS) between pairwise alignments were generated using the PAML algorithm (version 3.14; [43], [44]) using a codon-based model of sequence evolution [45], [46], with dN and dS as free parameters and average nucleotide frequencies estimated from the data at each codon position (F3×4 MG model [47]). For this analysis we assumed a transition/transversion bias (i.e. kappa value) that had been estimated previously and found to be similar in case of *P. falciparum* and *P. yoelii*, i.e. 1.53 [48]. A sliding window analysis of dN/dS ratios was performed of *p230*, *p47* and *p48/45* from the three rodent parasites. We analysed the dN/dS values of these genes across their length by analysing sequentially 300bp of the gene in 150bp steps. This analysis is essentially the same as the calculation of π (i.e. the number segregating or polymorphic sites) described for *p48/45* in distinct *P. falciparum* isolates described by Escalante *et al.* [29]. We obtained the single nucleotide polymorphisms (SNPs) data identified from field and laboratory isolates of *P. falciparum* (excluding all *P. reichenowi* SNPs) from PlasmoDB (www.PlasmoDB.org). The alignment of these SNPs along the different genes (to scale) was extracted from the Genome Browser page of PlasmoDB. The locations of the SNPs were aligned onto the schematic representation of the 6-cys genes of the rodent parasites. It should be noted that the alignment of the *p230 gene* of the different *Plasmodium* species was only possible around 1008bp after the putative start site. In order to determine which residues of *p230*, *p47* and *p48/45* genes were under positive selection in the rodent malaria parasites, a Bayes Empirical Bayes (BEB) analysis was performed using sequences from the 3 rodent genomes and was calculated as described in Yang *et al.* [49]. To test which genes were undergoing positive selection the likelihood ratio test (LRT) was performed using a comparison of site specific models of evolution [50], [51]. This test compares a ‘nearly neutral’ model (without any residues under positive selection) and a ‘positive selection’ model (with residues under positive selection and therefore under adaptive evolution). Both models assume that there are different categories of codons, which evolve with different speeds. The ‘nearly neutral’ model assumes two categories of sites at which amino acid replacements are either neutral (dN/dS = 1) or deleterious (dN/dS<1). The ‘positive-selection’ model assumes an additional category of positively selected sites at which non-synonymous substitutions occur at a higher rate than synonymous ones (dN/dS>1). Likelihood values indicate how well a model fits to the analyzed alignment and answers the question if the ‘positive selection’ model fits better to the analyzed alignment than the ‘nearly neutral’ model.

### Animal ethics statement

All animal experiments were performed after a positive recommendation of the Animal Experiments Committee of the LUMC (ADEC) was issued to the licensee. The Animal Experiment Committees are governed by section 18 of the Experiments on Animals Act and are registered by the Dutch Inspectorate for Health, Protection and Veterinary Public Health, which is part of the Ministry of Health, Welfare and Sport. The Dutch Experiments on Animal Act is established under European guidelines (EU directive no. 86/609/EEC regarding the Protection of Animals used for Experimental and Other Scientific Purposes).

## Results

### Four out of ten members of the 6-cys family of *P. berghei* are specifically transcribed in gametocytes

**Figure 1:**
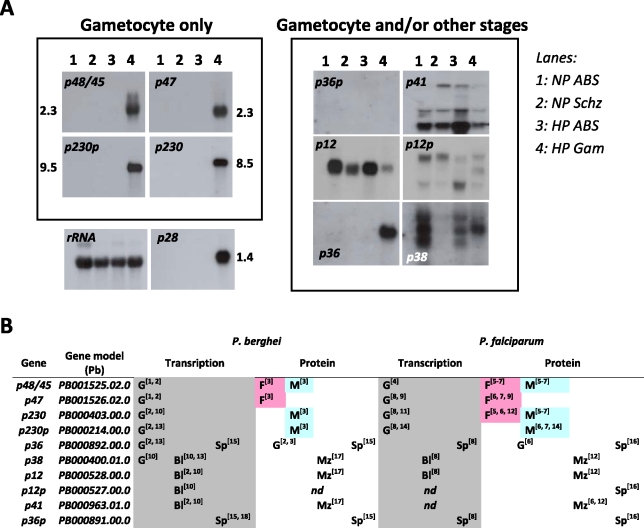
Expression of the 10 members of the 6-cys family of *Plasmodium*. **A.** Northern blot analysis of transcription of the 10 *P. berghei* genes during blood stage development of a gametocyte non-producer (NP) and a high producer (HP) line. The left panel shows the four genes that are exclusively expressed in gametocytes. *P36* and *p36p* are shown in the right panel since they are also expressed in the sporozoite stage (see B). As (loading) controls Northern blots were hybridized to probes recognising LSU rRNA (87R primer) and the gametocyte specific gene *p28*. Lanes: 1) NP asynchronous blood stages (ABS); 2) NP schizonts (Schz); 3) HP asynchronous blood stages; 4) HP purified gametocytes (Gam). **B.** Transcription and protein expression of the 10 genes determined by RNA and proteomic analyses (G = gametocyte; F = female gametocyte; M = Male gametocyte; Bl = blood stage; Mz = merozoite; Sp = sporozoite). References: 1 [4]; 2 [52]; 3 [17]; 4 [18]; 5 [71]; 6 [72]; 7 [73]; 8 [74]; 9 [12]; 10 [75]; 11 [10]; 12 [13]; 13 [8]; 14 [16]; 15 [14]; 16 [41]; 17 [76]; 18 [15].

Ten members of the 6-cys family have been identified in *Plasmodium* and are found in all *Plasmodium* species (Table S3). We analysed the transcription profile of the 10 members during blood stage development of *P. berghei* by Northern blot analysis and combined this analysis with a search of publicly available literature, transcriptome and proteome datasets. This method established that multiple members are transcribed in gametocytes of which four members, *p48/45*, *p47*, *p230*, *p230p*, are transcribed exclusively in the gametocyte stage (Fig. 1A). The gametocyte specific expression of *p48* and *p230p* has been shown before [4], [8]. Transcription of *p38* occurs both in gametocytes and in asexual blood stages as has also been reported [8], whereas *p12* is transcribed in all blood stages. The relative weak band observed in gametocytes might be due to low contamination of the gametocyte preparation with asexual blood stages (gametocyte samples always contain a small degree of contamination with schizonts when density gradients are used for gametocyte purification). Transcription of *p41* and *p12p* show a complex pattern of multiple transcripts in all blood stages. The close paralogue pair *p36* and *p36*p have quite different transcriptional profiles: *p36p* is not transcribed in blood stages but transcription is exclusive to sporozoites [14], [15] whereas *p36* is transcribed both in gametocytes (Fig. 1B; [8], [52]) and in sporozoites [14], [15].

Since no polyclonal or monoclonal antibodies exist for most of the 6-cys family members of *P. berghei*, except for P48/45 [4], P47 (this study) , P36 and P36p [14], data on expression of these proteins in different life cycle stages mainly comes from large-scale proteome analyses. For most members of the 6-cys family which have been detected by proteome analysis, the presence of the protein coincides with transcription of its gene (Fig. 1B). The exclusive presence of P48/45, P47, P230 and p230p in the proteomes of gametocytes corresponds to the transcription pattern of their respective genes. The presence of P48 and P47 in *P. berghei* gametocytes has been confirmed using polyclonal antibodies against these proteins (Fig. S1; [4]). P12, P38 and P41 have been detected in the proteome of merozoites which agrees with their transcription in the asexual blood stages and with their identification in the raft-like membrane proteome of the *P. falciparum* merozoite surface [13]. Also the presence of P36 in proteomes of both gametocytes and sporozoites [41], [52] and P36p in sporozoites [14], [41] fits with the transcription profile of these genes. Up to now only P12p has not been detected in any proteome of *Plasmodium*. Comparison of the transcription and expression patterns of the 10 conserved members of the 6-cys family of *P. berghei* with those of *P. falciparum* from large scale transcriptome and proteome analyses demonstrates that the expression patterns are conserved between the rodent and human parasite (Fig. 1B) and also confirms that four out of the 10 members are specific to the gametocyte stage.

### Three out of 4 members of the 6-cys family of *P. berghei* that are specifically transcribed in gametocytes play a role in fertilisation

**Figure 2:**
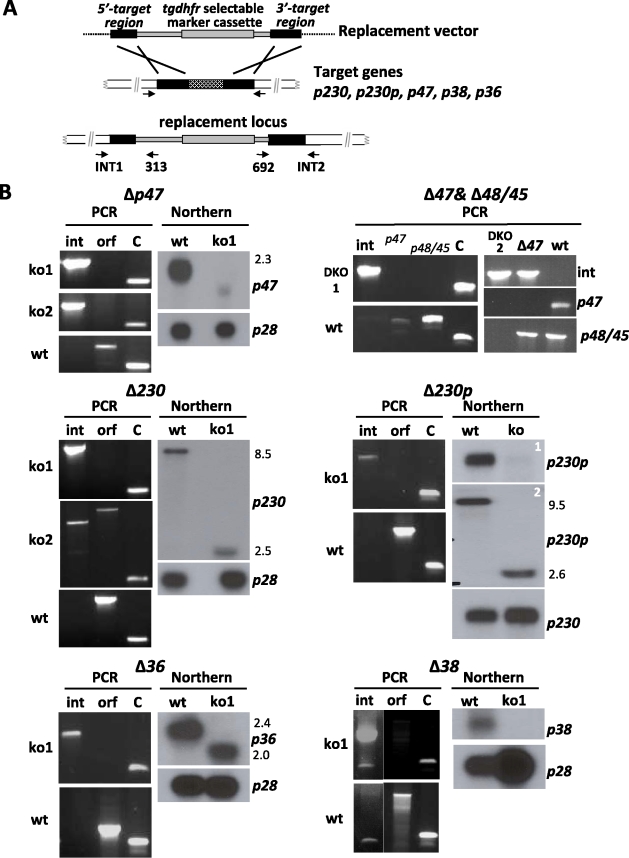
Generation and analysis of mutants lacking expression of different members of the 6-cys family of genes. **A.** Schematic representation of the replacement construct used for disruption of the target genes by double cross-over homologous recombination. Correct integration of the construct results in disruption of target gene as shown (replacement locus) and is analysed by PCR (see B) using the primers INT1, 313, INT2 and 692 as shown in the figure and Table S1 and S2. Black boxes: the target regions of the 6-cys genes; grey box: the *tgdhfr/ts* selectable marker cassette. **B.** PCR analysis of correct disruption of the 6-cys genes and analysis of transcription of the genes in wild type and mutant (ko) parasite lines. PCRs were performed with primers that specifically amplify either the 5′ (INT1 and 313) or 3′ (INT2 and 692) regions of the disrupted locus (int). In addition PCR's to amplify the intact open reading frame (orf) were performed using genomic DNA of wild type parasites as a control (wt). The double knockout mutant *Δp48*/45&*Δp47* was checked for both *p47* and *p48/45*. Control PCR amplifying the gametocyte specific *p28* gene (C). Northern blot analysis of transcription was performed using RNA extracted from gametocytes of wild type (wt) or mutant parasites. Blots were hybridised with 6-cys specific gene probes that were obtained by PCR amplification (see Table S2). As a control Northern blots were hybridized to a probe recognising the gametocyte specific gene *p28*. The sizes of transcripts (kb) are shown next to the Northern blots.

We previously reported the functional analysis of mutant *P. berghei* parasites that were deficient in expressing P48/45, generated by targeted disruption of *p48/45* through a double crossover homologous recombination event [4]. Here we have used the same approach, schematically shown in Fig. 2A, to disrupt 5 other members of the 6-cys family that are transcribed in gametocytes. We excluded *p12*, *p12p*, *p41* and *p36p* from this analysis since the results obtained from transcriptome and proteome analyses indicate a role for the first three of these genes during the asexual blood stage development (Fig. 1B). We have previously demonstrated in both, *P. berghei and P. falciparum*, that P36p is involved in liver-cell infection and disruption of its gene had no effect on development of gametes and fertilisation [15], [53]. Mutant parasite lines have been generated deficient in P47 (Δ*p47*), P230 (Δ*p230*), P230p (Δ*p230p*), P38 (Δ*p38*) or P36 (Δ*p36*) and for each gene, mutants were selected from two independent transfection experiments (Table S1). Two different *Δp230p* mutant lines were generated, Δ*p230p*-I and Δ*p230p*-II, differing in which regions of *230p* have been disrupted. In mutant Δ*p230*-I a fragment is deleted from the second 6-cys domain (i.e. first 894aa still present) onwards whereas in mutant Δ*p230*-II the deleted fragment includes part of the first 6-cys domain (i.e. first 492 amino acids still present). In addition we generated a mutant line deficient in the expression of both P48/45 and P47 (Δ*p48/45*&Δ*p47*). Correct disruption of the target-genes was verified by diagnostic PCR analysis (Fig. 2B) and Southern blot analysis of separated chromosomes and/or digested genomic DNA (data not shown). To demonstrate that the mutant parasite lines were deficient in expression of the targeted gene we analysed transcription of the corresponding genes by Northern blot analysis using mRNA collected from purified gametocytes (Fig. 2B). No transcripts of *p47* and *p38* could be detected in Δ*P47* and Δ*p38* mutants, and no *p48/45* and *p47* transcripts are present in the DKO mutant Δ*p48/45*&Δ*p47*. Only small, truncated transcripts were detected for *p230* and *p230p* in gametocytes of the Δ*p230* and Δ*p230p* lines and also in Δ*p36* a truncated p36 transcript was found. Full length transcripts of wt *p230* and *p230p* are 8.5 and 9.5 kb respectively, whereas truncated transcripts are approximately 2.5 kb in size. Since several of the disrupted genes are organised as pairs within the genome (i.e. *p230*&*p230*p and *p48/45*&*p47*), we analysed whether disruption of one member of a pair affected transcription of the other gene. For Δ*p48/45* parasites it has been shown before that disruption of *p48/45* had no effect on expression of its paralog P47 [4]. In this study we similarly show for *p47*, *p230* and *p230p* that disruption had no effect on transcription of its paralogous member (Fig. S1 A&B). In addition to the transcription analysis of the disrupted genes, we analysed the presence or absence of the proteins P47 and P48/45 in the mutant parasites by Western analysis using polyclonal antiserum (Fig. S1C). P47 is present in wt gametocytes and gametocytes of the Δ*p48/45* but is absent in Δ*p47* and Δ*p48/45*&Δ*p47* gametocytes. P48/45 is present in wild type and absent in the Δ*p48/45&*Δ*p47* gametocytes.

**Figure 3:**
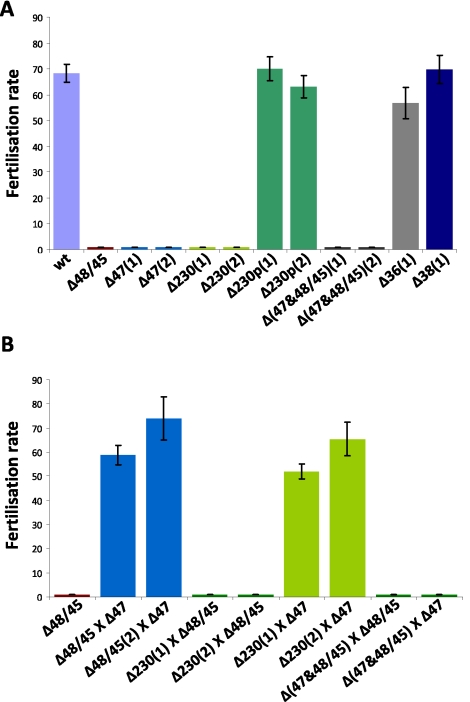
Fertilisation rates and male and female fertility of mutants lacking expression of different members of the 6-cys family of proteins. The fertilisation rate is defined as the percentage of female gametes that develop into mature ookinetes (ookinete conversion rates); 1 and 2 indicate mutants obtained from independent transfection experiments. **A.** Self-fertilisation rates of the different mutants, showing wild type fertilisation rates of mutants *Δp230p*, *Δp36 and Δp38*. **B.** Cross-fertilisation rates in assays in which gametes of the *Δp47*, *Δp230* and *Δp48/45& Δp47* mutants (that were affected in their fertilisation rate) were crossed with fertile females of *Δp48/45*. *Δp47* males are fertile and fertilise *Δp48/45* females at wild type rates whereas *Δp230* males are infertile. *Δp230* females are fertile and are fertilised by *Δp47* males at wild type levels. Gametes of both sexes of the *Δp48/45& Δp47* mutant are infertile.

We next analysed the phenotype of the different mutant lines during gametocyte and gamete development as well as during fertilisation, ookinete and oocyst formation using standard assays for phenotype analysis of the sexual- and mosquito stages of *P. berghei*. Surprisingly, three of the six mutants lacking expression of genes that are transcribed in gametocytes did not exhibit a phenotype that was different from wild type parasites during these stages of development. These mutants, Δ*p230p*, Δ*p38* or Δ*p36*, showed a normal growth of the asexual blood stage (data not shown), sexual development and development of the mosquito stages up to the mature oocysts (Table 1). All these mutant lines produced wild type numbers of gametocytes and gametes and showed normal fertilisation rates as measured by *in vitro* zygote/ookinete production (Table 1; Fig. 3). In contrast to the absence of a discernable fertilisation phenotype with the Δ*p230p*, Δ*p38* and Δ*p36* mutants, we found that the capacity of fertilisation is severely affected in the other three mutants, (Fig. 3A). Specifically, Δ*p47*, Δ*p230* and Δ*p48/45&*Δ*p47* lines showed a fertilisation rate that was reduced by more than 99.9% compared to wt, as shown by the inhibition of zygote/ookinete production *in vitro* (Table 1; Fig. 3A). These mutants produced normal numbers of mature gametocytes during blood stage development. The analysis of *in vitro* gamete formation (exflagellation of males; emergence of female gametes from the erythrocyte) by light-microscopy also revealed that the process of gametocyte and gamete formation was not affected, resulting in the production of motile male gametes and female gametes, emerged from the host erythrocyte by more than 80% of the mature gametocytes (Table 1). At 16–18h after activation of gamete formation, the *in vitro* cultures of Δ*p47*, Δ*p230* and Δ*p48/45&*Δ*p47* lines contained many (clusters of) unfertilized, singly nucleated, female gametes. This phenotype of a strong reduction of fertilisation despite the formation of male and female gametes closely resembles the phenotype of *Plasmodium* parasites lacking P48/45 [4]. As had also been previously observed with the P48/45 deficient mutant, the fertilisation rate of gametes of the three mutant lines seems to be more efficient in the mosquito compared to *in vitro* fertilisation [4]. Compared to wild type parasites, the *in vivo* fertilisation of the mutants is reduced by 93–98% as calculated by ookinete and oocyst production in mosquitoes (Table 1), whereas the reduction of *in vitro* fertilisation rate is greater than 99.9%. Infections of naïve mice through bite of 20–30 mosquitoes infected with parasites of Δ*p47*, Δ*p48/45*&Δ*p47*DKO and Δ*p230* parasites, resulted in blood stage infections containing only gene disruption mutants (i.e. mutant genotype and no ‘wild type’ parasites), as determined by PCR and Southern analysis of genomic DNA (results not shown). These results show that gametes of all three mutant lines still have a low capacity to fertilise, resulting in the production of viable and infective ookinetes, oocysts and sporozoites. Moreover, the results obtained with the double knock-out mutant *Δp48/45&Δp47* indicate that the few fertilisation events in single knock-out mutants deficient in expression of either P47 or P48/P45 (this study and [4]) cannot be explained by a compensation effect due to its paralogous protein because the *Δp48/45&Δp47* mutant still shows a comparable, albeit greatly reduced, ability to fertilise and to pass through the mosquito.

**Table 1:** Gametocyte/gamete production, fertilisation rate and development in mosquitoes of different mutants that lack expression of members of the 6-cys family of proteins.

Parasite	Gametocyte production^1^ % (SD)	Gamete production (%)^2^ ♂/♀	Fertilisation rate *in vitro* (%)^3^	No of ookinetes *in vivo* 4	No of oocyst^5^	Infected mosquitoes (%)
WT	19.9 (3.1)	86–94/89–96	59 (6.7)	1313 (293–4280)	298 (18–603)	100
Δ*p48/45&*Δ*p47*	20.7 (4.2)	82–94/84–94	<0.1	16 (0–78)	21 (0–124)	93
Δ*p48/45&*Δ*p47*	17.3 (2.1)	nd	<0.1	nd	nd	nd
Δ*p47* I	17.0 (2.0)	88–92/80–90	<0.1	50 (0–100)	16 (0–43)	95
Δ*p47* II	18.7 (2.5)	nd	<0.1	nd	17 (0–49)	70
Δ*p230* I	20.3 (3.2)	nd	<0.1	40 (0–100)	21 (0–76)	80
Δ*p230* II	18.3 (1.2)	84–96/82–86	<0.1	42 (0–100)	14 (0–59)	70
Δ*p230p* I	21.7 (2.5)	86–90/78–88	70.0 (4.6)	1320 (660–2060)	208 (26–579)	95
Δ*p230p* II	20.3 (1.5)	nd	63.0 (4.4)	nd	nd	nd
Δ*p36*	22.0 (1.7)	nd	56.7 (6.0)	nd	235 (18–563)	95
Δ*p38*	19.3 (2.3)	nd	69.7 (5.5)	nd	209 (20–556)	100

1 Percentage of blood stage parasites that develop into gametocytes in synchronous infections under standardized conditions.

2 Percentage of gametocytes that emerge from the host cell and form gametes, determined by counting exflagellations and free female gametes.

3 Fertilisation rate (FR) is the percentage of female gametes that develop within 18 hours into ookinetes *in vitro*.

4 Mean number and range of ookinetes per mosquito at 22 hours after mosquito feeding.

5 Mean number and range of mature oocysts per mosquito.

### P230 plays a role in male gamete fertility and P47 in female gamete fertility

Fertility of the male and female gametes produced by the mutant lines can be determined by *in vitro* cross-fertilisation studies, where gametes are cross-fertilised with gametes of parasite lines that produce either only fertile male gametes or female gametes. Such an approach was used to establish that Δ*p48/45* parasites produced infertile male gametes, whereas the female gametes are completely fertile [4]. We performed different *in vitro* cross fertilisation experiments to determine whether the reduced fertilisation capacity of the Δ*p47* and Δ*p230* mutants was due to affected male gametes, female gametes or to both sexes. Gametes of both mutants were cross-fertilised with female gametes of Δ*p48/45* (males are infertile) to determine male fertility of Δ*p47* and Δ*p230*. Male gametes of Δ*p47* were able to fertilise Δ*p48/45* females (at wild-type levels) whereas the males of Δ*p230* were unable to fertilise the Δ*p48/45* females (fertilisation rates <0.01%; Fig. 3B). These results demonstrate that male gametes of Δ*p47* are viable with wild type fertilisation capacity and therefore the fertilisation defect of Δ*p47* must be due to infertile females. The normal fertility of male gametes of Δ*p47* has also been shown in previous studies in which the males of this mutant have already been used in other cross-fertilisation studies [17], [39], [54], [55]. The lack of fertilisation in the crossing experiments of gametes of Δ*p230* with Δ*p48/45* shows that P230 plays a role in male fertility. In order to test the fertility of Δ*p230* females we crossed the gametes of this line with the fertile male gametes of Δ*p47* (as mentioned above the females are infertile). We find that Δ*p47* male gametes are able to fertilise Δ*p230* female gametes in a manner identical to their ability to fertilise Δ*p48/45* females (Fig. 3B). This demonstrates that female gametes of Δ*p230* have a fertility that is comparable to wild type female gametes and that the fertilisation defect is the result of infertile males. Crossing experiments performed with gametes of the double knockout mutant, Δ*p48/45&*Δ*p47* with gametes of either Δ*p230*, Δ*p47* or Δ*p48/45* did not result in increased fertilisation rates (<0.01%), demonstrating that gametes of both sexes are infertile in the double knock-out mutant (Fig. 3B).

### Infertile Δ*p230* males form exflagellation centres but do not attach to females and fertile males do not attach to infertile Δ*47 females*

In *P. falciparum* it has been shown that male gametes lacking P230 expression have a reduced capacity to adhere to red blood cells, as measured by the formation of ‘exflagellation centres’ [27]. We therefore examined the ability of *P. berghei* male Δ*p230* gametes to attach to erythrocytes, by microscopic examination of exflagellation centre formation under standardized *in vitro* conditions. In these experiments 76–92% of exflagellating wt males and 72–90% exflagellating Δ*p230* male gametocytes, formed such centres (Table 2), indicating that in contrast to *P. falciparum* Δ*p230* in *P. berghei* both wt and Δ*p230* male gametes have a similar ability to interact with red blood cells. Gametocytes that did not form exflagellation centres were often floating on/above the red blood cell layer during exflagellation. Further analysis of single, free male gametes of Δ*p230* revealed that they were highly motile and often attach to red blood cells, producing characteristic red blood cell shape deformations due to the active interactions between the male gamete and the erythrocyte. Male gametes lacking expression of P48/45 do not attach to female gametes as has been previously shown by analysing male-female interactions by light microscopy [4], [5]. We therefore analysed the interactions between male and female gametes of Δ*p230* or Δ*p47*, between 10 and 30 minutes after induction of gamete formation using phase-contrast microscopy. In wt parasites attachment of males to females was readily detected with a mean of over 25 attachments during a 20 minutes period of observation, with a mean of more than 6 confirmed fertilisations (i.e. male gamete penetrations; Table 2). In preparations of gametes of both Δ*p230* and Δ*p47* not a single fertilisation event was detected and the number of male and female gamete attachments was drastically reduced (Table 2). We observed that while male gametes of both mutants undergo active interactions with red blood cells and platelets, attachment of males to female gametes are hardly ever observed. These results show that P230 like P48/45 is a male fertility factor involved in recognition or attachment to females and that P47 is a female fertility factor involved in recognition or adherence by the male gamete. Whether P48/45 and P230 once on the surface of the male gamete directly interact with P47 on the surface of the female gamete is unknown. Unfortunately, repeated immuno-precipitation experiments with anti-*P. berghei* P48/45 antibodies and wt gamete preparations, in order to identify interacting partners, were unsuccessful (data not shown).

**Table 2:** The interactions of Δ*p230* and Δ*p47* male gametes with red blood cells (exflagellation centres) and female gametes (attachment and fertilisation).

	Exflagellation centers % of male gametocytes (range)	# of males attached to females (range)	# of fertilizations (range)
Wild type	84.7 (76–92)	25.5 (15–35)	6.8 (4–11)
Δ*p230*	80.3 (72–90)	2 (0–4)	0
Δ*p47*	nd	5.5 (2–8)	0

nd, not determined.

### Sequence polymorphism of *Plasmodium* proteins involved in fertilisation

Analyses of sequence polymorphisms of *p48/45*, *p47* and *p230* of laboratory and field isolates of *P. falciparum* has provided evidence that these proteins are under positive selection [28], [29], [30], [31]. We analysed synonymous (dN) and non-synonymous (dS) polymorphisms of *p48/45*, *p47* and *p230* by comparing these genes in three closely related rodent parasites *P. berghei*, *P. yoelii* and *P. chabaudi* by making use of the newly available gene sequences (www.PlasmoDB.org version 6.1). The updated dN/dS values for these genes obtained here, which is commonly used as an indicator of positive selection, were in all comparisons higher than the mean dN/dS value of all genes within the respective genomes (Table S4). However, only the dN/dS ratio of *p47* in the *P. berghei/P. yoelii* comparison showed a significant difference with the mean dN/dS value (0.82 compared to the mean dN/dS of 0.26). Overall, P47 is in the top 4–6% of fastest evolving proteins in the rodent parasite genomes as compared to top 10–16% for P230 and 15–50% for P48/45 (Table S4). In addition, we have used the likelihood ratio test (LRT) to analyse if these genes were undergoing neutral or positive selection (see Materials and Methods). This test shows that *p47* is indeed under positive selection (P = 0.006) when comparing the site/residue specific models of evolution.

**Figure 4:**
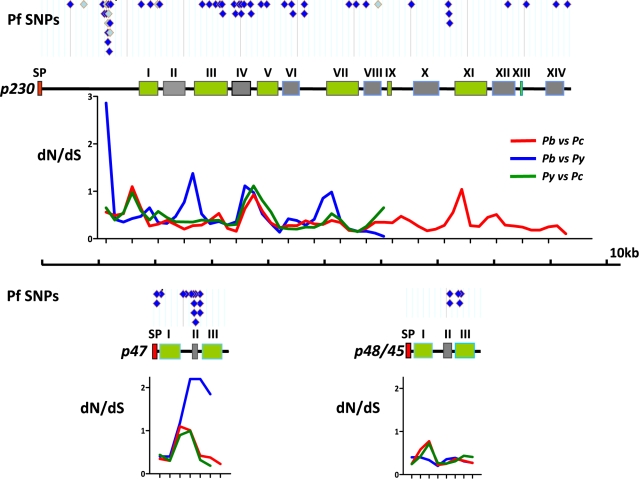
Polymorphisms and sequence divergence across *p230*, *p48/45* and *p47*. Schematic representation of *p230*, *p47* and *p48/45* (shown to scale). A- and B-type recurring domains (green and grey respectively; [7]) are shown and the numbering of domains (I–XIV) are shown as according to [7]. The putative Signal Peptide (SP) is indicated in red. Above each gene the locations of all single nucleotide polymorphisms (SNPs) are shown as identified in different *P. falciparum* strains in PlasmoDB (www.PlasmoDB.org; August 2009). Dark blue diamonds: non-synonymous polymorphisms; Light blue diamonds: synonymous polymorphisms. Below each gene the dN/dS ratios are shown across the length of the three rodent *Plasmodium* orthologs. This dN/dS analysis is performed using a ‘sliding-window’ analysis, where 300bp of corresponding DNA sequence was compared at 150bp intervals. The gene from each species has been compared to the same gene of the other species; Red: *P. berghei* against *P. chabaudi*; Blue: *P. berghei* against *P. yoelii*; Green: *P. yoelii* against *P. chabaudi*. The complete gene sequence is only available for *P. berghei* and *P. chabaudi*; The 5′ end of all three rodent parasite *p230* genes is shorter than those of the *P. falciparum p230 and* therefore alignment of the *P. falciparum* to the rodent *p230*s is only possible ∼1kb after the start site.

We next examined sequence mutations in the same genes in more detail by performing a comparative dN/dS ratio analysis across these genes using small and corresponding regions of these genes using a ‘sliding window analysis’ (i.e. 300bp in 150bp intervals; Fig. 4; Table S4). This analysis showed that *p47* has an exceptionally elevated dN/dS value (i.e. 1–2) in one area corresponding to the truncated B-type domain II as defined by [7]. Interestingly, although P230 had a relatively low overall dN/dS value (0.33–0.44), the sliding window analysis revealed that P230 contains several areas where the dN/dS ratio is higher than 1.0 with an increased ratio in all 3 species in particular around the B-type domain IV as defined by Gerloff *et al.* (2005). In order to analyse similarities in the location of sequence polymorphism between *P. falciparum* and the three rodent parasites, we aligned all known single nucleotide polymorphisms (SNPs) described for P230, P47 and P48/45 in *P. falciparum* (i.e. www.PlasmoDB.org; [56], [57], [58]) with the dN/dS ratios determined by the ‘sliding window analysis’ (for details see Materials and Methods; Fig. 4). Interestingly, the elevated dN/dS ratios of *p47* domain II and domain IV of P230, both correspond with the location of high SNP densities in the orthologous *P. falciparum* genes. These findings would suggest that similar regions in the *p47* and *p230* genes of rodent parasites and *P. falciparum* are subject to positive selection. To predict which residues of the three *P. berghei* genes are under positive selection we performed a Bayes Empirical Bayes analysis (BEB; [49]). This analysis calculates dN/dS values (ω values) on each residue of a particular protein when the genes encoding these proteins are compared in least 3 similar species and an ω>1 indicates positive selection on a residue. For P47 ten residues were identified undergoing positive selection with ω values ranging between 4 and 7 (Table S5). Nine of these 10 residues are confined to the first two domains of P47 including the region B-type domain II. In P48/45 four residues were identified (ω values ranging between 1 and 2) and for P230 only one amino acid (ω = 1.3). Interestingly, this one residue in P230 (i.e. residue 845V) maps to the corresponding region of the *P. falciparum* P230, domain IV, where 6 of the 27 non-synonymous polymorphisms described by Gerloff *et al.* map (Table S4).

## Discussion

Until recently the only protein proven to play a direct role in merging of the male and female gamete of *Plasmodium* gametes in *Plasmodium* was P48/45, a surface protein principally of male gametes shown to play an essential role in recognition of and attachment to females [4], [5]. Recently, two studies have identified a second protein, HAP2/GCS1 with a role early in fertilisation [5], [6]. Male gametes of mutant parasites lacking this protein can attach to female gametes but the subsequent fusion of the gametes is absent [5], a process which is clearly after the mutual recognition and attachment of gametes. Our studies provide evidence for the direct involvement of two additional proteins, P47 and P230, which like P48/45 play a key role in the initial phase of gamete-gamete recognition and attachment. The phenotype of mutants lacking P230 expression is identical to the phenotype of mutants lacking P48/45, i.e. male gametes do not recognize and attach to female gametes whereas the female gametes are fertile. These results show that the P230 protein, like P48/45, is a male fertility factor. A similar role of P48/45 and P230 in male fertility is perhaps not surprising since evidence has been reported that both proteins interact with each other. Unlike P48/45, P230 does not contain a glycosylphosphatidylinositol (GPI) anchor and in *P. falciparum* evidence has been found that P230 forms a complex with P48/45 at the surface of gametocytes and gametes [18], [27], [59], [60]. Indeed, analysis of *P. falciparum* mutants has shown that in the absence of P48/45 the P230 protein is not retained on the surface of gametes, a result which may indicate that tethering of P230 to the surface of the male gamete is mediated by P48/45 [27]. In contrast, in the absence of P230 the surface location of P48/45 is not affected in *P. falciparum* [27], [61]. If in *P. berghei* the same interaction occurs, and Δ*p48/45* gametes also lack surface expression of P230, then the failure of Δ*p48/45* and Δ*p230* males to attach to females might be solely due to the absence of P230 on the male gamete surface. This would imply that P230 and not P48/45 is the major male protein that is responsible for recognition of and attachment to the female. However, it has been shown that antibodies directed against P48/45 strongly reduce oocyst formation [19], [20], [24], [25], [26], indicating that either P48/45 antibodies disrupt the attachment of the translocated P230 to P48/45 after gamete formation or it may play a more direct role in fertilisation and that its function is not exclusively as a membrane anchor for P230.

Interestingly, in *P. falciparum* it has been shown that male gametes with a disrupted *p230* gene are incapable of interacting with erythrocytes and do not form the characteristic exflagellation centres and these mutants show a strong reduction in oocyst formation [27]. These observations, in *P. falciparum*, indicate that P230 not only plays a role in gamete-gamete interactions but male gamete interactions with erythrocytes may be required for gamete maturation resulting in an optimal fertilisation capacity [27], [62]. Our analyses of Δ*p230 P. berghei* male gametes in live preparations did not reveal any difference in their capacity to interact with red blood cells, suggesting that there are functional differences between P230 of *P. berghei* and *P. falciparum*. As the interaction between male and female gametes has not been analysed in the *P. falciparum* Δ*p230* mutants it is unknown whether the decreased oocyst formation results from the reduced gamete-erythrocyte interactions or is due to the lack of gamete recognition and attachment, as we have observed in *P. berghei*. Therefore, further research is needed to unravel whether *P. falciparum* P230 is also involved in gamete-gamete interactions like *P. berghei* P230. Moreover, additional research is required to identify the proteins at the surface of *P. berghei* male gametes that are responsible for the adherence of the male gametes to erythrocytes. Disruption of the close paralogue of *p230*, *p230p*, did not have any effect on fertilisation or on red blood cell attachment. The distinct phenotypes of Δ*p230* and Δ*p230p* gametes demonstrate that the proteins encoded by these genes are not functional paralogues that are able to complement each others function as has been demonstrated for the paralogous protein pair P28 and P25 on the surface of zygotes [63]. The same is true for the paralogous proteins P48/45 and P47 (see below) or P36 and P36p [15], [64].

In addition to the important role of P230 in male fertility, our studies demonstrate that P47 plays a key role in *P. berghei* female gamete fertility. Both proteome analyses of *P. berghei* gametocytes [17] and IFA analysis of *P. falciparum* gametocytes using anti-P47 antibodies [12] have shown the female-specific expression of P47. In *P. falciparum*, P47 is located on the surface of the female gametes following emergence from the host erythrocyte. Our studies demonstrate that *P. berghei* females lacking P47 are not recognized by wild type males. These observations may suggest that P48/45 or P230 on the male gamete directly interact with P47 on the female for recognition and attachment. However, P48/45 and P230 may alternatively interact with additional, as yet unknown protein/s on the surface of the female that are dependent on the presence of P47, in an analogous manner to the interaction between P230 and P48/45 on the surface of the male gamete. Both P48/45 and P230 are also expressed in the female gametes of *P. berghei* and *P. falciparum* [17], [27]. The presence of these proteins on the female gamete surface does not result from male proteins that are released by the male during activation and subsequent binding to the female since ‘pre-activated’ female gametocytes also express these proteins (B van Schaijk, personal communication and [65]. However, an essential role for P48/45 and P230 in female gametocytes is not implicated in *P. berghei* since both Δ*p230* and Δ*p48/45* females demonstrate normal fertilisation, i.e. to wild-type levels, when incubated with wild type males.

Unexpectedly, the lack of expression of P47 in *P. falciparum* mutants appears not to have a role in fertilisation as determined by oocyst formation in mosquitoes [12]. This difference between *P. berghei* and *P. falciparum* suggests that the proposed model of the interactions between male P48/45 and/or P230 with female P47 (and/or P47-interacting proteins) being key for the recognition and attachment of gametes does not hold true for all *Plasmodium* species. However, these differences between *P. falciparum* and *P. berghei* might also be explained by the presence of an additional set of protein ligands in both species that mediate additional mechanisms of gamete recognition and attachment. Indeed by analysing *P. berghei* Δ*p48/45* mutants [4] and mutants lacking expression of P47 and P230 (this study) we found that low levels of fertilisation did occur. Surprisingly, in all mutants significant higher fertilisation rates were observed in mosquito midguts compared to *in vitro* rates of fertilisation. Even in the mutant lacking expression of both P48/45 and P47, the same low fertilisation rates are observed. Assuming that *P. berghei* Δ*p48/45* gametes lack P230 surface expression as has been shown for *P. falciparum* Δ*p48/45*, then gametes of the double knock-out mutant can fertilise in the absence of essentially all three fertility factors of the 6-cys family, albeit at a reduced rate. These observations indicate the presence of additional proteins that secure fertilisation in the absence of the three members of the 6-cys family. For unidentified reasons this alternative fertilisation pathway appears to be much more efficient *in vivo* than *in vitro*, suggesting that mosquito factors influence this alternative route of fertilisation. The observed oocyst formation in Δ*p48/45* and Δ*p47 P. falciparum* parasites [4], [12] might therefore also be explained by this route of fertilisation and the presence of relatively high numbers of oocysts might indicate that this alternative pathway is more efficient in *P. falciparum* in *A. stephensi* compared to *P. berghei* in *A. stephensi*. Such alternative pathways of fertilisation may have implications for development of transmission blocking vaccines that block fertilisation using antibodies directed against members of the 6-cys family of proteins and therefore it is important to identify the additional proteins involved in the process of recognition and attachment of gametes. It is possible that other members of the 6-cys family that are expressed in gametocytes (P230p, P38 and P36) may be involved in the alternative pathways of fertilisation. Although we found that gametes lacking expression of these proteins did not show a significant reduction in fertilisation, the effect of their absence on gamete fertility may only become evident in the absence of P48/45, P47 and P230. Further research using mutants lacking multiple 6-cys members is required to reveal whether other 6-cys family members or other unrelated proteins play a role in alternative routes of fertilisation.

For P48/45, P47 and P230 in *P. falciparum* evidence has been published that these proteins are under differing rates of positive selection resulting in non-neutral sequence polymorphisms [28], [29], [30], [31]. Polymorphisms in gamete proteins may be a consequence of sexual selection as is the case for gamete proteins of other organisms [3], [66]. However, sequence polymorphism in these *Plasmodium* genes may also result from natural selection exerted by the adaptive immune system of the host. These three proteins are expressed in mature gametocytes, and as only a very small percentage of gametocytes ever get passed on to a mosquito, the vast majority of gametocyte proteins (including these 6-cys members) are eventually released into the hosts circulation where they are exposed to the host immune system. Indeed it has been shown that P48/45 and P230 both elicit humoral responses in infected individuals that can mediate transmission blocking immunity [22], [24], [67], [68], [69], [70]. Our analyses on dN/dS values of the three rodent parasites provide additional evidence that directional selection pressures affect sequence polymorphisms of gamete surface proteins, especially evident for the female specific *p47* which belongs to the top 4–6% fastest evolving genes in the rodent parasite genomes. Analysis of dN/dS variation across the genes by the sliding window approach on P230 identifies one region that is evolving rapidly in all the rodent parasites and, interestingly, this correlates with the same region in *P. falciparum* (B-type domain IV) that has the highest density of SNPs [7]. The correlation of the location of *P. falciparum* SNP's with increased dN/dS ratios in both P230 and P47 may indicate that similar selection pressures exists in different *Plasmodium* species. Whether this positive selection on these gamete proteins is driven by immune responses and/or mating interactions is presently unknown. However, insight into sequence polymorphisms in gamete surface proteins that are targets for TB vaccines and the influence of these polymorphisms on mating behaviour of parasites in natural populations of *P. falciparum* should help to improve TB vaccines development.

## Supporting Information

Table S1 Information on the replacement constructs used to disrupt the different members of the 6-cys gene family
(0.04 MB DOC)

Table S2 Information on primers used in PCR and Southern analysis in order to genotype the mutants with disrupted 6-cys genes
(0.04 MB DOC)

Table S3 Gene models of the different 6-cys gene family members in 6 *Plasmodium* species
(0.04 MB DOC)

Table S4 Whole gene dN/dS, dN and dS values of *p48/45*, *p47* and *p230* compared to the values of all annotated genes present in the 3 rodent parasite genomes
(0.04 MB DOC)

Table S5 Sliding window analysis of *p48/45*, *p47* and *p230* in *P. berghei* vs *P. yoelii* vs *P. chabaudi*
(0.07 MB PDF)

Table S6 Residues of P48/45, P47 and P230 under positive selection according to the Bayes Empirical Bayes (BEB) analysis
(0.07 MB PDF)

Figure S1 Gene expression of *p230*, *p47* and *p48/45* in mutants in which the paralogous gene has been disrupted. **A.** Northern analysis of transcription of *p230* and *p230p* in mutant *Δp230* showing wild type transcription of the paralog *p230p*. **B.** Northern analysis of transcription of *p47* and *p48/45* in the mutant *Δp47*, showing wild type transcription of the paralog *p48/45*. **C.** Western blot analysis of expression of P47 and P48/45 in mutants *Δp48/45* and *Δp48/45& Δp47*.
(0.10 MB PDF)

Figure S2 Gene alignments of *P. falciparum* and *P. berghei p230*, *p48/45* and *p47*. The one residue (861V) in *P. berghei p230* that appears to be under strong positive selection by the BEB analysis is highlighted (blue) and aligned with the two non-synonymous polymorphic residues in *P. falciparum* (i.e. 1194Y and 1196Q; in red and highlighted in yellow; defined by [7]) adjacent to a cysteine residue defined in domain IV of P230 (highlighted in yellow).
(0.05 MB PDF)
