# Supplementary material for: Three Members of the 6-cys Protein Family of Plasmodium Play a Role in Gamete Fertility
Source: PLoS Pathog. 2010 Apr 8;6(4):e1000853. doi: 10.1371/journal.ppat.1000853 (PMC2851734; doi:10.1371/journal.ppat.1000853)
Supplement: Table S1 — Information on the replacement constructs used to disrupt the different members of the 6-cys gene family (0.04 MB DOC) [file ppat.1000853.s001.doc]

**Table S1**: Information on the replacement constructs used to disrupt the different members of the 6-cys gene family

| **Gene** | **Construct** | **Mutant parasites** | **Primers**  **restriction sites** | **5'-targeting primers** | **Size (kb)** | **Primers**  **restriction sites** | **3'-targeting primers** | **Construct size (kb)** | **Insert release** |
| --- | --- | --- | --- | --- | --- | --- | --- | --- | --- |
| *p47* | pL1138 | 270cl1 526cl1 | L697 H,B  L701 H | cccaagcttggatccgattataatattccttcaataagg  cccaagcttgtaccttttccatatgctcatagtcg | 0.5 | L867 EV  L700 K | ggatatctagcaatgttggtggcattgc  cggggtaccaattttatcattagcgttatgtgg | 1.3 | B/K |
| *p47-*  *48/45* | pL0121 | 192cl1  203 | L697 H,B  L701 H | cccaagcttggatccgattataatattccttcaataagg  cccaagcttgtaccttttccatatgctcatagtcg | 0.5 | L408 EV  pBS-SK | ggatatcagcaatacctcaatcagcatc | 1.5 | B/K |
| *p230* | pL1139 | 310cl1 323cl1 | L925 H  L1360 H | cccaagcttcatattttcctaaaagagctcc  cccaagcttcacttttatatactatagcacc | 0.6 | L1357 EV  L1358 B | ggatatcggaaaatattttaatgaatctcc  cgcggatccgtatttctgaatgtggaattagc | 1 | C/B |
| *p230p I* | pL0122 | 204cl1  216 | L1.5 K  L1.6 H | cggggtaccgaaacaatcgaatttctatgc  cccaagcttttggcgtcccatctatgc | 0.42 | L1.3 EV  L1.4 B | agttcaaaaacaaattacacg  cgcggatcctactgtaataccttttttccc | 0.5 | K/B |
| *p230p II* | pL0120 | 314cl1 | L1345 H,S  L1346 H | cccaagcttccgcgggtatatggtaaagaacctactaacac  cccaagcttgatgtgttttatttggatgtgc | 0.66 | L1347 EI  L1348 S | ccggaattctcttgagcccgttaatg  tccccgcgggtatggaactacatctatatag | 0.9 | S |
| *p36* | pL0123 | 261cl1  276cl1 | L862 H,B  L863 H | cccaagcttggatccgcatttttgttgactctaccg  cccaagcttgactttttaatataactccaggc | 0.58 | L864 EV  L865 K | ggatatccgatttagcatctcatcatgg  cggggtacctggtactgcgaaaatcacacc | 0.9 | B/K |
| *p38* | pL0124 | 360cl1  846cl1 | L1355 C  L1356 H | ccatcgatatatttgtaaaatgagtgtgtgg  cccaagcttaccacgaacataatcttgtattc | 1 | L1431 EI  L1248 B | cggaattcggtgctgtaaactatagag  cgcggatccatgcgaagaaacgaaacactg | 0.8 | C/B |

B, *Ba*mHI; C, *Cla*I.; EI, *Eco*RI; EV, *Eco*RV; H, *Hin*dIII, K, *Kpn*I; S, *Sac*II
