# Supplementary material for: Three Members of the 6-cys Protein Family of Plasmodium Play a Role in Gamete Fertility
Source: PLoS Pathog. 2010 Apr 8;6(4):e1000853. doi: 10.1371/journal.ppat.1000853 (PMC2851734; doi:10.1371/journal.ppat.1000853)
Supplement: Table S2 — Information on primers used in PCR and Southern analysis in order to genotype the mutants with disrupted 6-cys genes (0.04 MB DOC) [file ppat.1000853.s002.doc]

**Table S2**: Information on primers used in PCR and Southern analysis in order to genotype the mutants with disrupted 6-cys genes

| **Disrupted**  **locus** | **INT1+2** | **Sequence** | **Size (kb)** | **WT1+2** | **Sequence** | **Size (kb)** |
| --- | --- | --- | --- | --- | --- | --- |
| *p47* | L759  L313 | atacagtaacgcaacgtcg  acgcattatatgagttcattttac | 1 | L964  L965 | actatgagcatatggaaaagg  cgcctaggctaggaagatgatatttttaattcc | 1 |
| *p47-48/45* | L759  L313 | agtacagtaacgcaacgtcg  acgcattatatgagttcattttac | 1 | L964  L965  L1384  L1385 | actatgagcatatggaaaagg  cgcctaggctaggaagatgatatttttaattcc  gctctagatgaaagaagatcagtaatatgtag  cgcggatccaccaattttaatattcataaaaccag | 1 (*p47*)  1.2 (*p48/45*) |
| *p230* | L1405  L313 | gatgtagaaccaagtgtagg  acgcattatatgagttcattttac | 1.5 | L1692  L1375 | cgcggatccacaggagataatacaaacaatgac  cgcggatccttattcaacaataccgattttcccattatc | 1.6 |
| *p230p I* | L831  L313 | ctttatttttcaattaccgcc  acgcattatatgagttcattttac | 1.3 | L932  L1359 | cccaagcttgaaacaatcgaatttctatgc  cccaagcttactgtaataccttttttccc | 1.6 |
| *p230p II* | L832  L692 | ttgtattcttcatcctcatatg  cttatatatttataccaattg | 1.5 | L831  L1348 | cccaagcttgaaacaatcgaatttctatgc  tccccgcgggtatggaactacatctatatagg | 1.6 |
| *p36* | L1000  L313 | tgcttatgcgtaaacaactcc  acgcattatatgagttcattttac | 1 | L1380  L1373 | cgcggatccgagtttaaaagaaatagaagttgg  cgcggatccttaatcttcttttgtggaaaaaatgtg | 0.9 |
| *p38* | L1210  L313 | taaagtgttacatacaatagttgc  acgcattatatgagttcattttac | 1.6 | L1355  L1248 | ccatcgatatatttgtaaaatgagtgtgtgg  cgcggatccatgcgaagaaacgaaacactg | 0.85 |
