# Supplementary material for: Three Members of the 6-cys Protein Family of Plasmodium Play a Role in Gamete Fertility
Source: PLoS Pathog. 2010 Apr 8;6(4):e1000853. doi: 10.1371/journal.ppat.1000853 (PMC2851734; doi:10.1371/journal.ppat.1000853)
Supplement: Table S3 — Gene models of the different 6-cys gene family members in 6 Plasmodium species (0.04 MB DOC) [file ppat.1000853.s003.doc]

**Table S3**: Gene models of the different 6-cys gene family members in 6 *Plasmodium* species

| **GENE** | ***P. falciparum*** | ***P. berghei*** | ***P. yoelii*** | ***P. chabaudi*** | ***P. vivax*** | ***P. knowlesi*** |
| --- | --- | --- | --- | --- | --- | --- |
| ***p48/45*** | PF13_0247 | PB001525.02.0 | PY04207 | PCAS_136420 | PVX_083235 | PKH_120750 |
| ***p47*** | PF13_0248 | PB001526.02.0 | PY04395 | PCAS_136430 | PVX_083240 | PKH_120710 |
| ***p36*** | PFD0210c | PB000892.00.0 | PY01341 | PCAS_100200 | PVX_001025 | PKH_031030 |
| ***p52*** | PFD0215c | PB000891.00.0 | PY01340 | PCAS_100210 | PVX_001020 | PKH_031020 |
| ***p12*** | PFF0615c | PB000528.00.0 | PY03100 | PCAS_011160 | PVX_113775 | PKH_113620 |
| ***p12p*** | PFF0620c | PB000527.00.0 | PY03099 | PCAS_011170 | PVX_113780 | PKH_113610 |
| ***p230p*** | PFB0400w | PB000214.00.0 | PY03857 | PCAS_030820 | PVX_003900 | PKH_041110 |
| ***p230*** | PFB0405w | PB000403.00.0 | PY03856 | PCAS_030830 | PVX_003905 | PKH_041100 |
| ***p38*** | PFE0395c | PB000400.01.0 | PY02738 | PCAS_110730 | PVX_097960 | PKH_102490 |
| ***p41*** | PFD0240c | PB000963.01.0 | PY01066 | PCAS_100250 | PVX_000995 | PKH_030970 |

.
