# Supplementary material for: Three Members of the 6-cys Protein Family of Plasmodium Play a Role in Gamete Fertility
Source: PLoS Pathog. 2010 Apr 8;6(4):e1000853. doi: 10.1371/journal.ppat.1000853 (PMC2851734; doi:10.1371/journal.ppat.1000853)
Supplement: Table S4 — Whole gene dN/dS, dN and dS values of p48/45, p47 and p230 compared to the values of all annotated genes present in the 3 rodent parasite genomes (0.04 MB DOC) [file ppat.1000853.s004.doc]

**Table S4**: Whole gene dN/dS, dN and dS values of *p48/45, p47* and *p230* compared to the values of all annotated genes present in the 3 rodent parasite genomes

|  | ***P. berghei vs P. yoelii*** | | | ***P. berghei vs P. chabaudi*** | | | ***P. yoelii vs P. chabaudi*** | | |
| --- | --- | --- | --- | --- | --- | --- | --- | --- | --- |
|  | dN/dS | dN | dS | dN/dS | dN | dS | dN/dS | dN | dS |
| *p48/45* | 0.36  (>79%) | 0.03 | 0.08 | 0.36  (>50%) | 0.05 | 0.14 | 0.36  (>85%) | 0.05 | 0.14 |
| *p47* | 0.82  (>96%) | 0.09 | 0.11 | 0.46  (>94%) | 0.09 | 0.19 | 0.50  (>94%) | 0.09 | 0.18 |
| *p230* | 0.44 (>87%) | 0.05 | 0.11 | 0.33  (>84%) | 0.09 | 0.26 | 0.42  (>90%) | 0.11 | 0.26 |
| All genes | 0.27 |  |  | 0.22 |  |  | 0.23 |  |  |
| All genes* | 0.26 |  |  | 0.22 |  |  | 0.23 |  |  |

* with telomeric multi-gene families excluded (e.g. birs, yirs, cirs etc)

Numbers in parentheses represent the percentage of genes within the *Plasmodium* genome that are evolving slower than the analyzed gene.
