# Supplementary material for: Three Members of the 6-cys Protein Family of Plasmodium Play a Role in Gamete Fertility
Source: PLoS Pathog. 2010 Apr 8;6(4):e1000853. doi: 10.1371/journal.ppat.1000853 (PMC2851734; doi:10.1371/journal.ppat.1000853)
Supplement: Table S5 — Sliding window analysis of p48/45, p47 and p230 in P. berghei vs P. yoelii vs P. chabaudi (0.07 MB PDF) [file ppat.1000853.s005.pdf]

Table S5

Sliding window analysis of *p48/45, p47* and *p230* in *P. berghei* vs *P. yoelii* vs *P. chabaudi*

PB000403.00.0 versus PY03856 versus PCAS_030830 (P230)

0	300	150	2.8624	0.561	0.6518
150	450	300	0.408	0.4908	0.3927
300	600	450	0.3521	0.5292	0.5516
450	750	600	0.4251	1.0976	0.973
600	900	750	0.4712	0.6501	0.5935
750	1050	900	0.6539	0.2689	0.396
900	1200	1050	0.3412	0.3097	0.5769
1050	1350	1200	0.3233	0.3854	0.4481
1200	1500	1350	0.4687	0.2996	0.3595
1350	1650	1500	0.7711	0.205	0.3569
1500	1800	1650	1.3756	0.2754	0.3525
1650	1950	1800	0.5234	0.2879	0.3933
1800	2100	1950	0.3188	0.3932	0.3782
1950	2250	2100	0.3625	0.5346	0.3921
2100	2400	2250	0.295	0.2199	0.2853
2250	2550	2400	0.3598	0.1582	0.3007
2400	2700	2550	1.1171	0.6329	0.8077
2550	2850	2700	0.9744	0.9292	1.1134
2700	3000	2850	0.5235	0.5876	0.8108
2850	3150	3000	0.3217	0.3347	0.5652
3000	3300	3150	0.1375	0.2323	0.2368
3150	3450	3300	0.4215	0.2798	0.2112
3300	3600	3450	0.383	0.2768	0.2026
3450	3750	3600	0.2868	0.379	0.2454
3600	3900	3750	0.4076	0.3133	0.2388
3750	4050	3900	0.853	0.3018	0.3227
3900	4200	4050	0.983	0.387	0.528
4050	4350	4200	0.3965	0.3431	0.4067
4200	4500	4350	0.2084	0.1756	0.2037
4350	4650	4500	0.1573	0.1561	0.1487
4500	4800	4650	0.159	0.2209	0.2577
4650	4950	4800	0.1178	0.3474	0.4543
4800	5100	4950	0.0516	0.3491	0.6539
4950	5250	5100		0.3368	
5100	5400	5250		0.4727	
5250	5550	5400		0.3825	
5400	5700	5550		0.2638	
5550	5850	5700		0.169	
5700	6000	5850		0.2002	
5850	6150	6000		0.2839	
6000	6300	6150		0.5564	
6150	6450	6300		1.0418	
6300	6600	6450		0.2757	
6450	6750	6600		0.2577	
6600	6900	6750		0.4509	
6750	7050	6900		0.5114	
6900	7200	7050		0.2897	
7050	7350	7200		0.2693	
7200	7500	7350		0.2472	
7350	7650	7500		0.1835	

7500	7800	7650		0.1819	
7650	7950	7800		0.2521	
7800	8100	7950		0.2725	
7950	8250	8010		0.1065	

PB001526.02.0 versus PY04395 versus PCAS_136430 (P47)

0	300	150	0.403	0.3466	0.4373
150	450	300	0.3993	0.3002	0.3035
300	600	450	1.185	1.0989	0.896
450	750	600	2.1968	1.0067	0.9921
600	900	750	2.1969	0.4173	0.3191
750	1050	900	1.844	0.3747	0.185
900	1200	1050		0.225	

PB001525.02.0 versus PY04207 versus PCAS_136420 (P48/45)

0	300	150	0.4005	0.2499	0.244
150	450	300	0.4015	0.5824	0.4231
300	600	450	0.3361	0.7737	0.7185
450	750	600	0.2007	0.224	0.2664
600	900	750	0.3559	0.2507	0.2553
750	1050	900	0.3878	0.3634	0.3094
900	1200	1050	0.3075	0.3222	0.4356
1050	1350	1200	0.2723	0.2683	0.4075
