# Supplementary material for: Three Members of the 6-cys Protein Family of Plasmodium Play a Role in Gamete Fertility
Source: PLoS Pathog. 2010 Apr 8;6(4):e1000853. doi: 10.1371/journal.ppat.1000853 (PMC2851734; doi:10.1371/journal.ppat.1000853)
Supplement: Table S6 — Residues of P48/45, P47 and P230 under positive selection according to the Bayes Empirical Bayes (BEB) analysis (0.07 MB PDF) [file ppat.1000853.s006.pdf]

Residues of P48/45, P47 and P230 under positive selection according Bayes Empirical Bayes (BEB) analysis

Protein ID	residue	P-value (omega>1)	omega	
PB000403.00.0				***p230***
	859V	0.518	1.28	
PB001526.02.0				***p47***
	6G	0.872	6.95	
	24F	0.811	6.46	
	29V	0.979	7.58	
	76N	0.792	6.26	
	79E	0.603	4.96	
	152R	0.809	6.44	
	160E	0.813	6.45	
	162I	0.857	6.75	
	183Q	0.632	5.10	
	233S	0.526	4.33	
PB001525.02.0				***p48/45***
	118T	0.5	1.47	
	204F	0.501	1.47	
	211D	0.544	1.56	
	339S	0.53	1.52	

Each *P. berghei* protein is compared to its ortholog in *P. yoelii* and *P. chabaudi*
