# Supplementary figures and images for: Three Members of the 6-cys Protein Family of Plasmodium Play a Role in Gamete Fertility
Source: PLoS Pathog. 2010 Apr 8;6(4):e1000853. doi: 10.1371/journal.ppat.1000853 (PMC2851734; doi:10.1371/journal.ppat.1000853)

**A**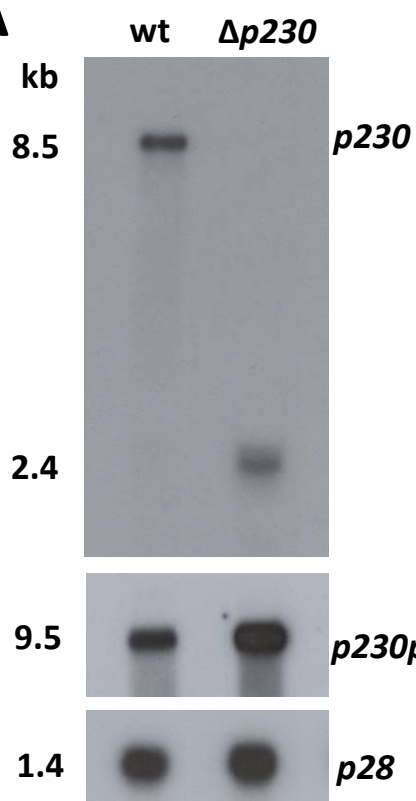**B**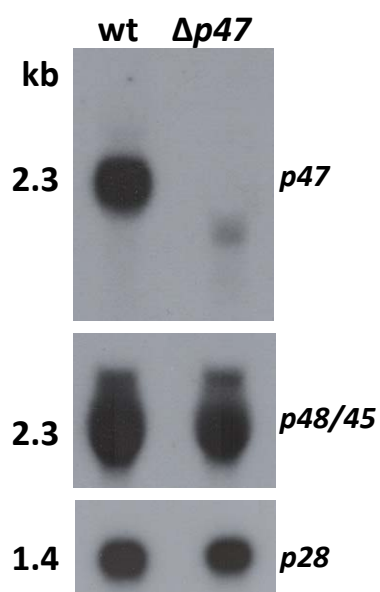**C**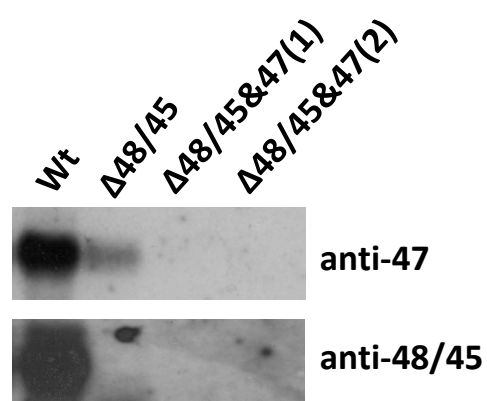

Supplement: Figure S1 — Gene expression of p230, p47 and p48/45 in mutants in which the paralogous gene has been disrupted. A. Northern analysis of transcription of p230 and p230p in mutant Δp230 showing wild type transcription of the paralog p230p. B. Northern analysis of transcription of p47 and p48/45 in the mutant Δp47, showing wild type transcription of the paralog p48/45. C. Western blot analysis of expression of P47 and P48/45 in mutants Δp48/45 and Δp48/45& Δp47. (0.10 MB PDF) [file ppat.1000853.s007.pdf]
