# Supplementary material for: Three Members of the 6-cys Protein Family of Plasmodium Play a Role in Gamete Fertility
Source: PLoS Pathog. 2010 Apr 8;6(4):e1000853. doi: 10.1371/journal.ppat.1000853 (PMC2851734; doi:10.1371/journal.ppat.1000853)
Supplement: Figure S2 — Gene alignments of P. falciparum and P. berghei p230, p48/45 and p47. The one residue (861V) in P. berghei p230 that appears to be under strong positive selection by the BEB analysis is highlighted (blue) and aligned with the two non-synonymous polymorphic residues in P. falciparum (i.e. 1194Y and 1196Q; in red and highlighted in yellow; defined by [7]) adjacent to a cysteine residue defined in domain IV of P230 (highlighted in yellow). (0.05 MB PDF) [file ppat.1000853.s008.pdf]

P48/45 global alignment

```
PB001525.02.0      1 -MLYFFGNSRFFLFFFYFFFYFVLVIKSSVGKNEYVSPDELNIKTSGFLG     49
                      |||.........    |..|.|||::...|.|::..|..||.:.|||:|
PF13_0247          1 MMLYISAKKAQVA----FILYIVLVLRIISGNNDFCKPSSLNSEISGFIG     46


PB001525.02.0     50 YKCDFSTEGIHNLEPDIVERRSVICSINSYFIYDKIKLIIPKQDDPKSKF     99
                     |||:||.||:|||:||:.||||:.|:|:||||||||:|||||:.. ..:|
PF13_0247         47 YKCNFSNEGVHNLKPDMRERRSIFCTIHSYFIYDKIRLIIPKKSS-SPEF     95


PB001525.02.0    100 KLLPENCFAKVYSDIEGKTEIPIEQTGLIEYTLEENDTNQDYSERIIQIS    149
                     |:|||.||.|||:|.|.:.|..|.:.|||||.:||||||.:|:||.|.||
PF13_0247         96 KILPEKCFQKVYTDYENRVETDISELGLIEYEIEENDTNPNYNERTITIS    145


PB001525.02.0    150 PFNNKDIEFYCICDNTEQVISHIDGRSALVHVTVLKYPHKIVSVNLTDQK    199
                     ||:.|||||:|.|||||:|||.|:||||:|||.||||||.|:..|||:..
PF13_0247        146 PFSPKDIEFFCFCDNTEKVISSIEGRSAMVHVRVLKYPHNILFTNLTNDL    195


PB001525.02.0    200 YPYLFDAYNKNDFINYKLEIGLKEGELLVLACKQIDNKCFQKNDESKNGD    249
                     :.||...||:::|::..||:.|.:|||.||||:.|:.||||   |.|...
PF13_0247        196 FTYLPKTYNESNFVSNVLEVELNDGELFVLACELINKKCFQ---EGKEKA    242


PB001525.02.0    250 LYKTNKIIYHKDFAIFKAPIYVKSNNATAECKCKIDEANIYTLVVKPDYD    299
                     |||:|||||||:..|||||.||.|.:...||.||....| |.:|:||.|:
PF13_0247        243 LYKSNKIIYHKNLTIFKAPFYVTSKDVNTECTCKFKNNN-YKIVLKPKYE    291


PB001525.02.0    300 EKVIYGCNFSKDLSFR-TFTNNMNLLKYNENTNINCNVEISQPFYDHLIG    348
                     :|||:|||||.::|.: |||::::...::::.:|:|||.:|:|.|:||:|
PF13_0247        292 KKVIHGCNFSSNVSSKHTFTDSLDISLVDDSAHISCNVHLSEPKYNHLVG    341


PB001525.02.0    349 ISCPGTIIPDCFFQIYKPLTNELKSSEITYLDSQLNIGNIEYYEDIHGNN    398
                     ::|||.||||||||:|:|.:.||:.|.|.|||||:|||:||||||..|::
PF13_0247        342 LNCPGDIIPDCFFQVYQPESEELEPSNIVYLDSQINIGDIEYYEDAEGDD    391
```

```
PB001525.02.0    399 EIRIFSIVGAIPQSASFTCMCKMDKITGFMNIKIGSAYYAFLSKLFIIFI    448
                     :|::|.|||:||::.||||:||.||.:.:|.:.|.||||.||:|.||..|
PF13_0247        392 KIKLFGIVGSIPKTTSFTCICKKDKKSAYMTVTIDSAYYGFLAKTFIFLI    441

PB001525.02.0    449 PLFFMWL    455
                     ....:::
PF13_0247        442 VAILLYI    448
```

p47 global alignment

```
PB001526.02.0      1 ---MKGFTGASIIVFYL---IKGYLSYIIFPNGYVCDFKFNPLVNVLPSI     44
                        .:..:..:||:||.   :|..:|.::....|||||.||||.||.|::
                   1 MCMGRMISIINIILFYFFLWVKKSISELLSSTQYVCDFYFNPLTNVKPTV     50

PB001526.02.0     45 NTTGDI-EEVGCTINNPSLSDYIALVCPKKNYNDYEHMEKVPSKCFSSNL     93
                     ..:.:| ||||||||||:|.|:|.|:|||||..|:.::|.||:.||.|:|
                  51 VGSSEIYEEVGCTINNPTLGDHIVLICPKKNNGDFSNIEIVPTNCFESHL    100

PB001526.02.0     94 YSPYKSEDSAHKLEELKIPEKYSISKDFSDFDLNIILIPSLYNIDKTIYC    143
                     ||.||::.||:.||:|.|.:||:|:..||||.|.|::||:.|...|||||
                 101 YSAYKNDSSAYHLEKLDIDKKYAINSSFSDFYLKILVIPNEYKSHKTIYC    150

PB001526.02.0    144 RCDNSKTKRELIKNDGENIKLQGKLGLVKIILNNQQNSPQNIYHITRSTQ    193
                     |||||||::.:   .|::..|:||||||||||.||.|   ||..:.::..
                 151 RCDNSKTEKNI---PGQDKILKGKLGLVKIILRNQYN---NIIELEKTKP    194

PB001526.02.0    194 VGSLDNKV------IELKEGEIVHLKYDGKTRT---NFNCKEIINMKISI    234
                     :  :.||.      |:|||.:|  |.:..|..|   :.||:||:|.||::
                 195 I--IHNKKDTYKYDIKLKESDI--LMFYMKEETIVESGNCEEILNTKINL    240

PB001526.02.0    235 PLDYNLSMRMPTVFLKDINCKFHLIFSNVGGIANIV-FKAKKTENIDGCD    283
                     ..:.|:.::||::|:.:|||.  |...:.....|.: .||.||::|||||
                 241 LSNNNVVIKMPSIFINNINCM--LSSQDQNNEKNYINLKADKTKHIDGCD    288
```

```
PB001526.02.0    284 FTVPKGKGLYKNGFALSEINNDEE-ICTVHIGRGQNSNAAGLKCPYNLTP    332
                     ||.|||||:|||||.:::|.|:|| |||||:...:|...||:||||.|.|
                 289 FTKPKGKGIYKNGFIINDIPNEEERICTVHLWNKKNQTIAGIKCPYKLIP    338

PB001526.02.0    333 AHCFKHVLYEKKYKNGNNVFQTFLLDDVLRTVDIEYYYNAKLSAHIVGIP    382
                     .:|||||||||:. :....::||||.|||.|.:||||.|.|...:::.:|
                 339 PYCFKHVLYEKEI-DSQKTYKTFLLSDVLDTPNIEYYGNNKEGMYMLALP    387

PB001526.02.0    383 TIPEKSETVRCVCEHDGKKGIMELKISSSKNIFISFILLSIIVSIFYLF-    431
                     |.|||:..:||:||..|||.:|||.|:|:...:||..|:..::.|||::
                 388 TKPEKTNKIRCICEQGGKKAVMELHIASTSTKYISMFLIFFLIVIFYMYV    437

PB001526.02.0    431 --    431

                 438 SI    439

Local alignment p230
We used the Waterman algorithm and amino acid positions 1-200 are not aligned

PB000403.00.0    184 DSEYDEDDEDDEDDEDDEK---PGAESDVEPSVGSDEEPYEESDEVIEND    230
                     |.||.|..|..||.|:..|   ...:|...||||.||
PFB0405w         520 DGEYGEYGEAVEDGENVIKIIRSVLQSGALPSVGVDE-------------    556

PB000403.00.0    231 INIDQINETYELAIDKNIFV-EGRKGNFTGDNTNNDYVCDFRKHIKVSES    279
                       :|:|:.:||.....:..| |.....:..:|||.:|||||...:|.:||
PFB0405w         557 --LDKIDLSYETTESGDTAVSEDSYDKYASNNTNKEYVCDFTDQLKPTES    604

PB000403.00.0    280 KESEKVCELKIVKPIANINIICPTKDLGNGEFENITYFPKRAPYIVLELT    329
                     ....|.||:|:.:|:..:.||||.|......::||.|.||::||:|  ||
PFB0405w         605 GPKVKKCEVKVNEPLIKVKIICPLKGSVEKLYDNIEYVPKKSPYVV--LT    652

PB000403.00.0    330 KKTEKESQTKLKEKKIADLLYGTIIPKIHNGKNNNFNRGSIRFILPPIVV    379
                     |:     :||||||.::.|:||.:|....|.|.|||..|.|.|.|||:|.
PFB0405w         653 KE-----ETKLKEKLLSKLIYGLLISPTVNEKENNFKEGVIEFTLPPVVH    697
```

```
PB000403.00.0    380 KPTTFYIICDNSRSKKNSFRGKKGIVKIHVAPYGNIEKGCNY--SKNKNH    427
                     |.|.||.|||||:::.::.:|.:|||:::|.||||...||.:  ...:..
PFB0405w         698 KATVFYFICDNSKTEDDNKKGNRGIVEVYVEPYGNKINGCAFLDEDEEEE    747

PB000403.00.0    428 LFGININNYEKEKQIDEIE---------NRKGNL-PCFFQLNAGEAGGII    467
                     .:|   |..|:::..::|:         .:|.|: ||:.:|.:|:.|||:
PFB0405w         748 KYG---NQIEEDEHNEKIKMKTFFTQNIYKKNNIYPCYMKLYSGDIGGIL    794

PB000403.00.0    468 FPENYKSSTCFEEVVEYNPNKNWNRKKKEINELINGAIVYKSDIKYKYFN    517
                     ||:|.||:|||||::.||....||::.|.:..|:|.::||..::..||||
PFB0405w         795 FPKNIKSTTCFEEMIPYNKEIKWNKENKSLGNLVNNSVVYNKEMNAKYFN    844

PB000403.00.0    518 AIYIGIPTSYRKSLHIFCNITTTD------NSFHMVYLSINQEINISVFD    561
                     ..|:.|||||:.:|::||:|...:      ::.::||:|||:|:|.|:||
PFB0405w         845 VQYVHIPTSYKDTLNLFCSIILKEEESNLISTSYLVYVSINEELNFSLFD    894

PB000403.00.0    562 LFESFIRLNKMTEVAQK-VDQK-NEYICDFTKRLDAPHDNNENNKKVIIC    609
                     .:|||:.:.|..:|||| |:.| ::|.||||.:||....:..|.||:.||
PFB0405w         895 FYESFVPIKKTIQVAQKNVNNKEHDYTCDFTDKLDKTVPSTANGKKLFIC    944

PB000403.00.0    610 RKKLKEFDIFRLKCDFNKTKNKNIGMIPGSLKENQHVLKLDLDVQFLLLK    659
                     ||.|||||.|.|||:.|||:..||.:.|.:||:.:.|||||||:|:.:..
PFB0405w         945 RKHLKEFDTFTLKCNVNKTQYPNIEIFPKTLKDKKEVLKLDLDIQYQMFS    994

PB000403.00.0    660 LYMKLNSKLYKYVHEYPPLFIFPLNIISKLELKKNIIFKNHKYGKYFNES    709
                     .:.|.|::..||::.||...|||.|.|.|.|||.|..:||||..|||.:|
PFB0405w         995 KFFKFNTQNAKYLNLYPYYLIFPFNHIGKKELKNNPTYKNHKDVKYFEQS   1044

PB000403.00.0    710 PNKNGL-----LVNLLTYLNSLDVLSMNTEIESINIDVND--SDQNIVDI    752
                     ...:.|     |..||.:|::.:.:.:..:|..:|:.:|:  ||.|...:
PFB0405w        1045 SVLSPLSSADSLGKLLNFLDTQETVCLTEKIRYLNLSINELGSDNNTFSV   1094

PB000403.00.0    753 SFQVPAYIYNTNPYYFVFGCNNTRDNGKIGIVELIISKNEEMIKGCNFNS    802
```

```
                           :||||.||....|:||:|||||.:..|.||||||:|||.||.||||||:.
PFB0405w           1095 TFQVPPYIDIKEPFYFMFGCNNNKGEGNIGIVELLISKQEEKIKGCNFHE   1144

                       □
PB000403.00.0       803 DAIEHFSNNMRPDETECKIDAYPNDIIGFICPKKQNFVSSKHVLDIDADT    852
                        ..:::|:.|:..|..||.:.||.||||||.|      :.:.|..::::.:.
PFB0405w           1145 SKLDYFNENISSDTHECTLHAYENDIIGFNC------LETTHPNEVEVEV   1188
```

Domain IV (B-type)

```
PB000403.00.0       853 DADLENVDVNPNDCFDSINIDSTKKYIVNELPGAQTYRNKSRNMPRYFKV    902
                        : |.| :.:.|.:||:::.........|...|..||||...::..|.:.|:
PFB0405w           1189 E-DAE-IYLQPENCFNNVYKGLNSVDITTILKNAQTYNINNKKTPTFLKI   1236

PB000403.00.0       903 PYHNNELDVIFQCSCVMGSKTNKIIVTVKALNGQIPKKYEKSEIKSSPSI    952
                        |.:|...||...|.|.:......||.|.:....:..:.|:..:||     |.
PFB0405w           1237 PPYNLLEDVEISCQCTIKQVVKKIKVIITKNDTVLLKREVQSE-----ST   1281

PB000403.00.0       953 VDGPVRACPHKQSIEPKLIKWNNKDTETIDSCKIDVNRFYSYAQLACPNN   1002
                        :|..:..|.|:..|.|::.|..:::.|.  :|.|.:..|::|.|:.||..
PFB0405w           1282 LDDKIYKCEHENFINPRVNKTFDENVEY--TCNIKIENFFNYIQIFCPAK   1329

PB000403.00.0      1003 NFSMHSNIQLTYNILKPNKIDEYKTFNESELNNLIPHSEILVDVVES---   1049
                        :..::.|||:.|:|:||.::.::|.||..||:.|||:||:|....|.
PFB0405w           1330 DLGIYKNIQMYYDIVKPTRVPQFKKFNNEELHKLIPNSEMLHKTKEMLIL   1379

PB000403.00.0      1050 NNDINVDTYNLYLFFPYYIKENYEINVLCDNTNTTYESKKGGKYIYNIKI   1099
                        .|:..||..:.|:|.|.|||:.||.|::|||:.|.::::.|||.||:|.:
PFB0405w           1380 YNEEKVDLLHFYVFLPIYIKDIYEFNIVCDNSKTMWKNQLGGKVIYHITV   1429

PB000403.00.0      1100 PKREKKVKGCNFNSNKKSEMFENGEIIKLDENTNTCKIDAKPKDVIAFEC   1149
                        .|||:|||||:|: |:.:.||...:      .|...|.|||||||:|.|.|
PFB0405w           1430 SKREQKVKGCSFD-NEHAHMFSYNK-----TNVKNCIIDAKPKDLIGFVC   1473

PB000403.00.0      1150 PPETIKITNCFKDAIIDNKLTNISTILQLNNNISSWTYGHKTSYLEVPAV   1199
                        |..|:|:||||||||:...||||:.||.|.||::::||.|:.:|:|:||:
```

```
PFB0405w        1474 PSGTLKLTNCFKDAIVHTNLTNINGILYLKNNLANFTYKHQFNYMEIPAL   1523

PB000403.00.0   1200 IDADATLTCICVDL-KKIYDIKSVFSPKFLNEIYKKLGIANGIYTKPILS   1248
                     :|.|.:..|||||| ||.|::||...||.|..:||||.|....|......
PFB0405w        1524 MDNDISFKCICVDLKKKKYNVKSPLGPKVLRALYKKLNIKFDNYVTGTDQ   1573

PB000403.00.0   1249 HKSIKYNSKFFEKYKENKIGNFFDFIKNTKIAEPDSDPQNIIENLFIENF   1298
                     :|.:.........:|.|.:...|..:...|.|:.|::|::|||:|.|...
PFB0405w        1574 NKYLMTYMDLHLSHKRNYLKELFHDLGKKKPADTDANPESIIESLSINES   1623

PB000403.00.0   1299 DKY----TSNIKQDEYYLDKYSVLNGFDVWENTYDKVLEETIIKDIESFE   1344
                     ::.    |.::..:      :.:|.|:|.||:.||:.|||.|..||||.|
PFB0405w        1624 NESGPFPTGDVDAE------HLILEGYDTWESLYDEQLEEVIYNDIESLE   1667

PB000403.00.0   1345 KRNFKIYTLEVSLKSSKLVKPIKLADNKYVCDFSKNNLIVPE-----KEI   1389
                     .::.:.|.|:|:||:.||:...::.:|::|||||||||||||     :|:
PFB0405w        1668 LKDIEQYVLQVNLKAPKLMMSAQIHNNRHVCDFSKNNLIVPESLKKKEEL   1717

PB000403.00.0   1390 GNIPKDVNCYTILSPLDTLYVKCPTIKSKYSEAEREIEDEDDEYEKEVIE   1439
                     |..|.:::||.:|.|||||||||||.|..|..|:..|.:.|:|||.:||.
PFB0405w        1718 GGNPVNIHCYALLKPLDTLYVKCPTSKDNYEAAKVNISENDNEYELQVIS   1767

PB000403.00.0   1440 LIDRL---------------DVPEIESDVETDPNVLLDKEKYTKYFNKLT   1474
                     ||::.               ||......|:|.|  :||...:.|||..:.
PFB0405w        1768 LIEKRFHNFETLESKKPGNGDVVVHNGVVDTGP--VLDNSTFEKYFKNIK   1815

PB000403.00.0   1475 LKPKSFFSSVTNYLYDVEEK--TETVLKGAIFTEMKVLKKSNPYISYASI   1522
                     :||..||..|.|...|.||:  .|::|.|||.:.||||||.:|:.|||:.
PFB0405w        1816 IKPDKFFEKVINEYDDTEEEKDLESILPGAIVSPMKVLKKKDPFTSYAAF   1865

PB000403.00.0   1523 IIPPFIESNEILKVECDNTRYK-EGGNIAGYNGIAYIELSKHKSKIKGCD   1571
                     ::||.:..:...||||:||.|| |...|:|||||.:|::|....||.|||
PFB0405w        1866 VVPPIVPKDLHFKVECNNTEYKDENQYISGYNGIIHIDISNSNRKINGCD   1915
```

```
PB000403.00.0   1572 FTQHESTILTKGTNSVE-KDETCNIDVYSNEPFGIMCSSSYSLEPKNCFY   1620
                     |:.:.|:|||.....|. :.:.|.|::.:||.|||:|.:..:|:|:.||:
PFB0405w        1916 FSTNNSSILTSSVKLVNGETKNCEININNNEVFGIICDNETNLDPEKCFH   1965

PB000403.00.0   1621 KIYDKIN-NVKNFKELIPNATILSLTNS-KQVVAYAKVPSDYMYKLKFSC   1668
                     :||.|.| .||.|:|:|||..|.||.|| |:.|||||||.||:.||.|||
PFB0405w        1966 EIYSKDNKTVKKFREVIPNIDIFSLHNSNKKKVAYAKVPLDYINKLLFSC   2015

PB000403.00.0   1669 KCKGKY-NKEKTVNILLNNGELEYDDLNIVNSIKANNINLCNFFENNELS   1717
                     .||..: |...|:.:.||..|.|.:|......||.||::|||||:|.||:
PFB0405w        2016 SCKTSHTNTIGTMKVTLNKDEKEEEDFKTAQGIKHNNVHLCNFFDNPELT   2065

PB000403.00.0   1718 LLNTPNKIVLCEINPDLFSEVIALFPILGEENSEQ----PKYKKYNTTPE   1763
                     ..|  ||||||:|:.:||||||...||.|.:|.|:    .:|||::..|.
PFB0405w        2066 FDN--NKIVLCKIDAELFSEVIIQLPIFGTKNVEEGVQNEEYKKFSLKPS   2113

PB000403.00.0   1764 LEVDSN---IKFIGDDKIKHTLSSILKGVIGNRIFNFKKIKQTVDGQTKG   1810
                     |..|.|   ||.||.:|.:.::|..||||.|||||.|.|     :|: ||
PFB0405w        2114 LVFDDNNNDIKVIGKEKNEVSISLALKGVYGNRIFTFDK-----NGK-KG   2157

PB000403.00.0   1811 IGFSFIVPPVLENINLKFQINEKTDVESVLKPRGLIYIFIKKNVDPNILK   1860
                     .|.||.:||:.::.:|||.|||..| .|.:|.|||||||::|||..|..|
PFB0405w        2158 EGISFFIPPIKQDTDLKFIINETID-NSNIKQRGLIYIFVRKNVSENSFK   2206

PB000403.00.0   1861 VCDFTSGKINLIGSNVHNLEKKCNINIRGGDIFGIICPKGFTLFPKGCFS   1910
                     :||||:|..:|:..|....||||.:.|:.|||||:.|||||.:||:.|||
PFB0405w        2207 LCDFTTGSTSLMELNSQVKEKKCTVKIKKGDIFGLKCPKGFAIFPQACFS   2256

PB000403.00.0   1911 SVILEYYNNSNHEKYENIMNTKNYMNSVQEIIYNPKPKEIKELLDEGYND   1960
                     :|:||||.:.    ||:..:...|::..::  ||.|||::.||:||.:.:
PFB0405w        2257 NVLLEYYKSD----YEDSEHINYYIHKDKK--YNLKPKDVIELMDENFRE   2300

PB000403.00.0   1961 LEDMENFSNFSNITEILNFKNYNMGNMKWDYNKYRSSSYAQVPETFDTIL   2010
```

```
                        |::::.::..||||::|:|||:|:||:..::..:.|::||:||:||::|:
PFB0405w          2301 LQNIQQYTGISNITDVLHFKNFNLGNLPLNFKNHYSTAYAKVPDTFNSII   2350

PB000403.00.0     2011 KFSCSCYNPQNEISGLMTVQSENIDLANKKTDQTIYENNIILPYKNKVNK   2060
                       .|||:||||:..:.|.|.|:|:|.:..|.|.::.:.: |.:||
PFB0405w          2351 NFSCNCYNPEKHVYGTMQVESDNRNFDNIKKNENVIK-NFLLP-------   2392

PB000403.00.0     2061 NLEAVSI----------------------------NKNNYYDNQNDEYFY   2082
                       |:|..::                            ::.:.:|:.|..:.|
PFB0405w          2393 NIEKYALLLDDEERQKKIKQQQEEEQQEQILKDQDDRLSRHDDYNKNHTY   2442

PB000403.00.0     2083 KPDSIIEKSNHYICDY-SDEFFFNFV---EKKIKRNICKINANKFDIITV   2128
                           |:..||.:|||| .:|...:.:   .|||:::||||||...|::|:
PFB0405w          2443 ----ILYDSNEHICDYEKNESLISTLPNDTKKIQKSICKINAKALDVVTI   2488

PB000403.00.0     2129 KCPYTKNVNSQKIEKEQDVSTNIENEIIIYFDDTEYITYFNETDKTVSLK   2178
                       |||:|||...:.......:.|| :.:|:|.||...::||.:.|.||.|||
PFB0405w          2489 KCPHTKNFTPKDYFPNSSLITN-DKKIVITFDKKNFVTYIDPTKKTFSLK   2537

PB000403.00.0     2179 DIYLKKFYGRAKEELQELNILSINPENNNIYY-PKNILNDVIINNQIVKL   2227
                       |||::.|||.:.:.|.::..:....::.:::| |.|:|::|::||.||.|
PFB0405w          2538 DIYIQSFYGVSLDHLNQIKKIHEEWDDVHLFYPPHNVLHNVVLNNHIVNL   2587

PB000403.00.0     2228 QSALPGVIFLNNK-TDDK---DENNQLQTDGESTFIIPPFIKKDFHFQVI   2273
                       .|||.||:|:.:| |.|:   .:|..|.|||.|:.:|||::|:|..|.:.
PFB0405w          2588 SSALEGVLFMKSKVTGDETATKKNTTLPTDGVSSILIPPYVKEDITFHLF   2637

PB000403.00.0     2274 CGKYITEN-SKESISLGIVYINISANLNQINGCDFI-----------NPE   2311
                       |||..|:. :|::.||.:::|:||:|.|.|:||||:           |..
PFB0405w          2638 CGKSTTKKPNKKNTSLALIHIHISSNRNIIHGCDFLYLENQTNDAISNNN   2687

PB000403.00.0     2312 NSSDSIFIHKKNETNNLICEIELVPNRIVGINCPNKKLKPENCFSQMYYI   2361
                       |:|.|||.|.||..|||||:|.|:|..::||.||||||.|:.||.::||:
PFB0405w          2688 NNSYSIFTHNKNTENNLICDISLIPKTVIGIKCPNKKLNPQTCFDEVYYV   2737
```

```
PB000403.00.0   2362 NESEFNGSIELLNDSTN---KVDITNLIKNTVSINNIKRSNNTYSYLILP   2408
                     .:.:. .|..:..|..|   |..|.|::||.:||||....:|||:|||||
PFB0405w        2738 KQEDV-PSKTITADKYNTFSKDKIGNILKNAISINNPDEKDNTYTYLILP   2786


PB000403.00.0   2409 NYIDLDANID--KLFICTCNDKYIAKIKTDP---ESIKAENKHKTETRYC   2453
                     ...: :..||  |:..|||::|||..:|.:.   :.||.:.|.......|
PFB0405w        2787 EKFE-EELIDTKKVLACTCDNKYIIHMKIEKSTMDKIKIDEKKTIGKDIC   2835


PB000403.00.0   2454 EYDYINKISRCNIMEGMETNVSDINSAVITYYS-SLSRWDRLIIKYPTSN   2502
                     :||...|::.|.|::.::::|...:..|  :|| :|||||:|||||||:.
PFB0405w        2836 KYDVTTKVATCEIIDTIDSSVLKEHHTV--HYSITLSRWDKLIIKYPTNE   2883


PB000403.00.0   2503 KFLFESSFVNPFNLKEKVLYN-NMPTYIDDILPGAIIYNKYDARTKLIEY   2551
                     |..||:.||||||||:||||| |.|..|:.||||||..:.||.|||:.:|
PFB0405w        2884 KTHFENFFVNPFNLKDKVLYNYNKPINIEHILPGAITTDIYDTRTKIKQY   2933


PB000403.00.0   2552 TLRIPPYVPKHIQFSIEFNNRYTLTNYNEEKVQGNVAYINVNVNQGHKEI   2601
                     .|||||||.|.|.||:||||..:||..|:..:.||||.|.:::|||:|||
PFB0405w        2934 ILRIPPYVHKDIHFSLEFNNSLSLTKQNQNIIYGNVAKIFIHINQGYKEI   2983


PB000403.00.0   2602 NGCDFTGKYSNLFTKSFNSITHETKECTIHFTTNNKFAGFACPSNYNIKP   2651
                     :|||||||||:|||.|...:.::...|.:.. .||.|:||||.|::.:||
PFB0405w        2984 HGCDFTGKYSHLFTYSKKPLPNDDDICNVTI-GNNTFSGFACLSHFELKP   3032


PB000403.00.0   2652 NSCFANIYDNKDSQKVKKLSEISANAEYDYIKYNSKGYTLSYVTFNNENK   2701
                     |:||:::||..::.|||||.::|...|.|:||.|:.||||||:.||.|:.
PFB0405w        3033 NNCFSSVYDYNEANKVKKLFDLSTKVELDHIKQNTSGYTLSYIIFNKEST   3082


PB000403.00.0   2702 EQNISCQCIGENSTYTINIIFEP-FSPRQPMSILRPFIKYVHLKPGTFNK   2750
                     :...||.|....|.|||.|.|:| :...:|.|  |..||||.|:...|.|
PFB0405w        3083 KLKFSCTCSSNYSNYTIRITFDPNYIIPEPQS--RAIIKYVDLQDKNFAK   3130
```

```
PB000403.00.0   2751 YLRR   2754
                     |||:
PFB0405w        3131 YLRK   3134
```
